# Explainable Deep Learning Framework for SERS Bioquantification

**DOI:** 10.1021/acssensors.5c01058

**Published:** 2025-09-02

**Authors:** Jihan K. Zaki, Jakub Tomasik, Jade A. McCune, Sabine Bahn, Pietro Lió, Oren A. Scherman

**Affiliations:** † Melville Laboratory for Polymer Synthesis, Yusuf Hamied Department of Chemistry, 2152 University of Cambridge, Lensfield Rd, Cambridge CB2 1EW, U.K.; ‡ Department of Chemical Engineering and Biotechnology, 2152 University of Cambridge, Philippa Fawcett Drive, Cambridge CB3 0AS, U.K.; § Department of Computer Science and Technology, University of Cambridge, 15 JJ Thomson Ave, Cambridge CB3 0FD, U.K.

**Keywords:** SERS, deep learning, denoising autoencoder, explainable AI, serotonin, biomarker quantification, CRIME, urine analysis

## Abstract

Surface-enhanced Raman spectroscopy (SERS) is rapidly gaining attention as a fast and inexpensive method of biomarker quantification, which can be combined with deep learning to elucidate complex biomarker-disease relationships. Current standard practices in SERS analysis are behind the state-of-the-art machine learning approaches; however, the present challenges of SERS analysis could be effectively addressed with a robust computational framework. Furthermore, there is a need for improved model explainability for SERS analysis, which at present is insufficient in assessing the contexts in which confounding factors affect prediction outcomes. This study presents a framework for SERS bioquantification rooted in a three-step process, including spectral processing, quantification, and explainability. A serotonin quantification task in urine was assessed as a model task, with 682 SERS spectra measured in a micromolar range using cucurbit[8]­uril chemical spacers. A denoising autoencoder was utilized for spectral enhancement, while convolutional neural networks (CNNs) and vision transformers were utilized for biomarker quantification. In addition, a context representative interpretable model explanation (CRIME) method was developed to suit the current needs of SERS mixture analysis explainability. Serotonin quantification was most efficient in denoised spectra analyzed using a CNN with a three-parameter logistic output layer (mean absolute error = 0.15 μM, mean percentage error = 4.67%). Subsequently, the CRIME method revealed the CNN model to present six unique prediction contexts, of which three were associated with serotonin. The proposed framework could unlock a novel, untargeted hypothesis-generating method of biomarker discovery, considering the rapid and inexpensive nature of SERS measurements and the potential to identify biomarkers from CRIME contexts.

## Graphical abstract

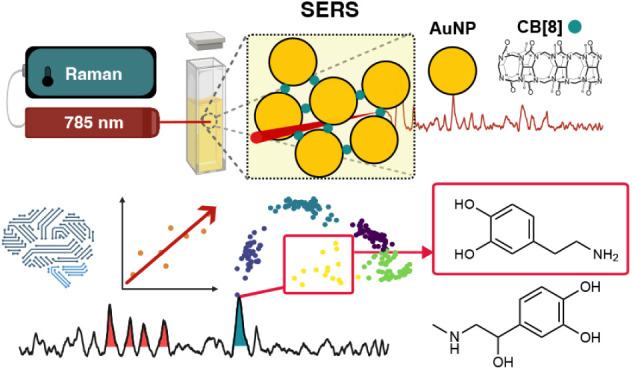

## Introduction

Deep learning methods are increasingly being used in biomarker research, as yet-to-be-discovered relationships between biomarkers and disease outcomes increase in complexity with the expanding number of biomarkers investigated.^1^ Surface-enhanced Raman spectroscopy (SERS) presents a novel molecular assaying method and allows for the delivery of consistent, accurate, and sizable data that can be utilized with deep learning methods. The technique capitalizes on “hot spots”, localized regions of intense optical fields created by the aggregation of noble metal nanoparticles.^2,3^ These nanoparticles offer a robust platform for *in situ* analysis within liquid media, rendering SERS a practical choice for broad applications.^4^ There are a set number of challenges in SERS-based analyte detection, which, if solved, could unlock the significant potential of the method. These primarily relate to the reproducibility, and readability of spectra, particularly in mixtures.^5^ Namely, inherent variability in SERS affects the signal intensities of spectra in repeat measurements, and the complexity of biological media measured, alongside potential *intra*- and cross-individual variation in molecular patterns, clouds the spectra through biological noise.^6^ Specifically related to biological noise, biomarker media almost universally involve significant numbers of confounding factors. Examples of such confounders could be other analytes of similar chemical structure in quantification tasks or clinical confounders that correlate with prediction outcomes in medical classification tasks. A significant headwind exists within the SERS domain as long as these challenges remain, as experiments are hard to verify as robust if confounding factors cannot be methodologically ruled out. While experimental developments are seeing improvements in the applicability of SERS, computational frameworks must be developed to supplement and enable the application of SERS in clinical practice.

The SERS domain is far behind current state-of-the-art practices developed in the field of machine learning, and several promising methodologies have yet to be integrated into SERS analyses. SERS analysis has relied on traditional dimensionality reduction methods to reduce the variations of the spectra and to account for the high noise levels of SERS spectra, particularly in biological samples. Principal component analysis and discriminant analysis (PCA-DA) and partial least-squares regression (PLSR) have been the *de facto* industry standard within the SERS domain;^4,7–12^ however, while these methods can be useful for feature extraction, the increased complexity of biological media can hinder predictions. Over the last 5 years, the application of convolutional neural networks (CNNs)^13^ for SERS analysis has become more common.^14–17^ Nevertheless, the application of CNNs has been predominantly applied with established model architectures with limited exploration of domain-specific modified layers. Moreover, although transformers have revolutionized various domains of machine learning by enabling models to handle sequential data with long-range dependencies effectively, their application in SERS spectral analysis remains underexplored. To date, we were able to source only two studies where vision transformer models (ViTs)^18^ were applied in SERS-based applications.^19,20^ Therefore, further assessing their utility against an established methodology within the SERS domain is crucial. Similarly to transformer models, the application of autoencoders in the field of SERS analysis is scarce and is primarily deployed for improved feature extraction^21,22^ despite their strong promise as robust denoising models. Most notably, a majority of SERS studies fail to show adequate model explainability. Without significant exploration of the models’ selected features, it cannot be determined without reasonable doubt that the predictions are due to the intended signal, confounders, or other sources of sample bias.

This study aims to develop computational methods to mitigate the described primary challenges of SERS. These are the variability between spectra, the effects of biological noise on measurements, and the difficulty in identifying confounding factors that prevent models from differentiating target analyte signals. To this end, the present study proposes a complete and up-to-date SERS analysis framework enabling robust bioquantification and explainability. The assessment of urinary serotonin was selected as a model task to develop our methodology. Urine is noninvasive, easy to obtain in large quantities, and shows significant potential as a biomarker source, with over 5000 analytes identified to date.^23^ In turn, serotonin plays a crucial role in regulating mood, emotion, and sleep, among other physiological functions. Imbalances in serotonin levels have been closely associated with carcinoid tumors,^24,25^ and it has long been hypothesized to be a causal factor in major depressive disorder (MDD), anxiety, and schizophrenia.^26–30^ In brief, this study aims to extend the clinical applicability of SERS in three main directions. First, it seeks to computationally mitigate inherent biological and method-based variations in SERS. Second, it aims to explore deep learning models for more accurately targeting analyte quantification. Lastly, it aims to propose a framework for explaining the decision-making process of the developed models, which could identify all contexts in which a model uses different predictors to reach the desired target outcome.

## Methods

### Data Set Preparation

The study design is summarized in Figure 1. The data set assessed consisted of 318 SERS spectra measured in a lyophilized urine medium and 364 SERS spectra measured in a water medium. Samples were measured using a 785 nm laser and cucurbit[8]­uril (CB[8]) spacers (0.9 nm) with 60 nm gold nanoparticles^4,31–33^ as visualized in Figure 1A. Cucurbit­[n]­urils are macrocyclic molecules consisting of glycoluril monomers, which act as a form of molecular “glue”, enabling controlled aggregation of the gold nanoparticles. Their primary utility is in facilitating improved consistency across spectral measurements through creating exact cross-nanoparticle distances for the plasmonic “hot-spots”.^34^ Both urine and water-medium samples were spiked with three key neurotransmitters: epinephrine, dopamine, and serotonin, with concentrations ranging from 0 to 9 μM. Serotonin was used as the target analyte. This was due to the lyophilized urine medium containing varying endogenous concentrations of epinephrine and dopamine, resulting in their unknown absolute concentrations. Furthermore, the serotonin concentrations evaluated reflect clinically relevant concentrations, with typical concentrations ranging between 0 and 2 μM, and the remaining concentrations representing abnormalities, such as carcinoid tumors.^24,25^ The specific peaks of serotonin, as well as their band assignments, are presented in Supplementary Figure 1 and Supplementary Table 1, respectively. For comparison, reference peaks for both dopamine and epinephrine are presented in Supplementary Figures 2 and 3. The SERS spectra were shortened to feature a relevant range of Raman shifts from 300 to 2000 cm^–1^, resulting in a total of 842 data points per spectrum. The specific concentrations of the neurotransmitters in each sample are listed in Table 1. Prior to assessment, spectra were processed using the asymmetric least-squares (ALS) algorithm^35^ for baseline correction with prespecified parameters (λ = 1000, *p* = 0.1, *n* = 10). Following the correction, the data was normalized to intensities between 0 and 1.

**Table 1:** Added Concentrations and Number of Spectra for All Three Neurotransmitters in Both Water and Urine Backgrounds^a^

Sample	**EPI** (μM)	**DA** (μM)	**5-HT** (μM)	**Water** (n)	**Urine** (n)
A	2	0	0	25	22
B	0	2	0	17	21
C	0	0	2	22	22
D	3	0	7	0^b^	26
E	0	8	3	0^b^	28
F	7	3	0	0^b^	21
G	3	2	7	99	26
H	1	1	9	38	22
I	2	8	3	37	22
J	6	9	1	93	23
K	7	3	2	11	23
L	9	6	4	11	21
M	3	3	3	11	0^b^
U	0	0	0	58	41

a EPI = epinephrine, DA = dopamine, 5-HT = serotonin, and *n* = number of spectra.

b Samples D, E, and F were not present in the water dataset, and sample M was not present in the urine dataset. Sample U represents a baseline measurement with no added or measured concentrations of the neurotransmitters.

**Figure 1:**
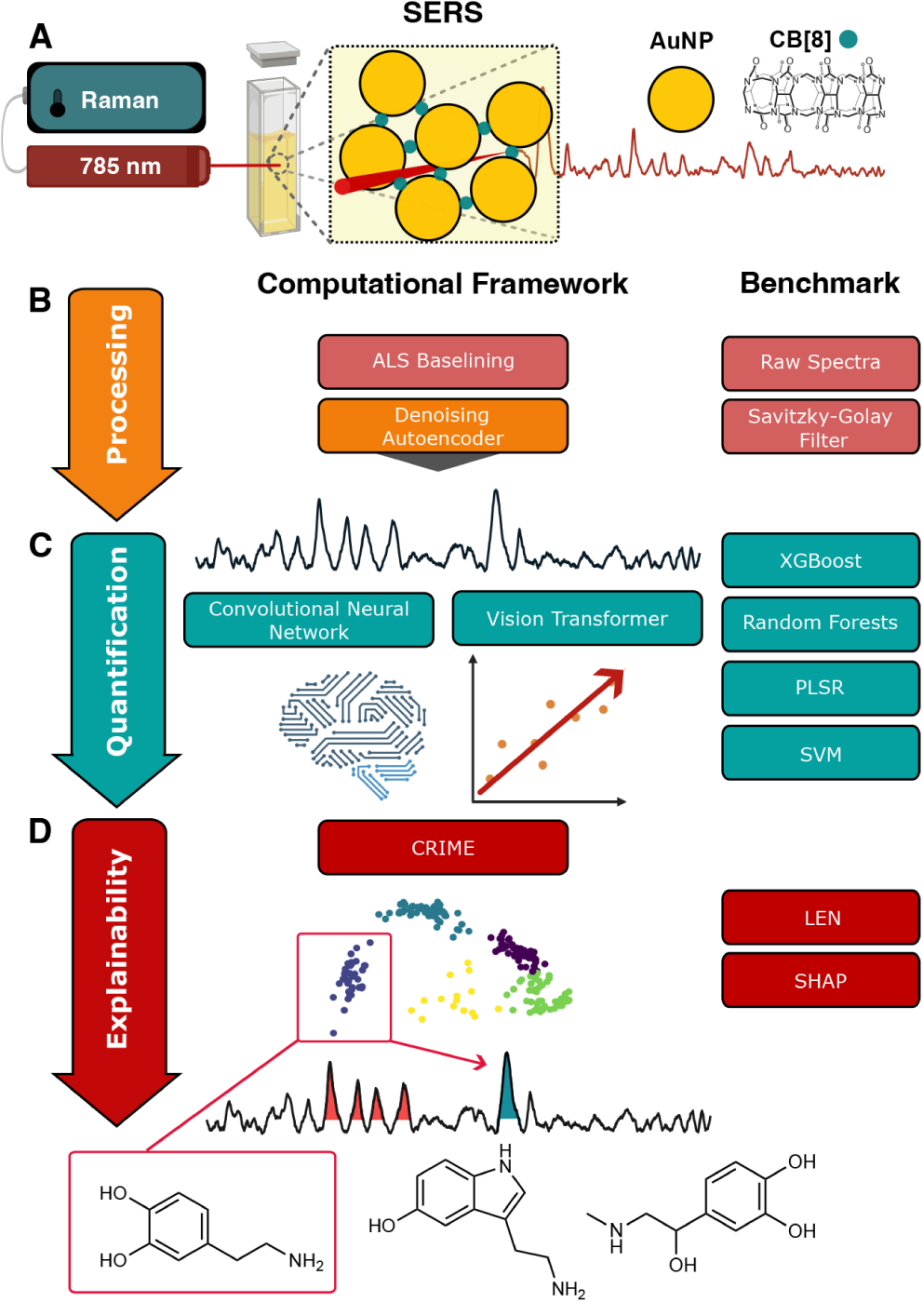
SERS deep learning framework development pipeline. Illustrated are the SERS measurement process applied (A) and the computational framework pipeline. Benchmark comparisons of alternative methodology are presented on the right. Preprocessing methods (B) are marked in orange and light red, quantification methods (C) are marked in blue, and explainability methods (D) are marked in dark red. Asymmetric least-squares (ALS) basislining is applied to all spectra prior to assessing the framework or the benchmarks. SERS = surface-enhanced Raman spectroscopy, AuNP = gold nanoparticle, CB[8] = cucurbit[8]­uril, CNN = convolutional neural network, XGBoost = extreme gradient boosting trees, PLSR = partial least-squares regression, SVM = support vector machines, CRIME = context representative interpretable model explanations, LEN = logic explained networks, SHAP = Shapley additive explanations.

### Denoising Autoencoder

Following baseline correction and normalization, the spectra were denoised by using a denoising autoencoder. For conventional autoencoders, an encoder neural network is trained to convolute input data into a latent space, and simultaneously, a decoder neural network is trained to restructure the original data from the latent transformation. A denoising autoencoder differs by attempting to reconstruct clean outputs from a latent space formed by encoding noisy data,^36^ which could prove useful in SERS applications with significant biological noise. Biological noise in the present study is defined as the SERS signals and background arising from analytes in the sample that are not the target analyte (i.e., serotonin). A simple denoising autoencoder was trained using full water-medium spectra consisting of 364 spectra with 937 data points using an 80:20 train-test split, with the urine background samples incorporated as noise. Noisy data were generated using urine background data (Table 1: sample U), where a randomly selected measurement was overlaid to each clean spectrum following baseline correction but before normalization. The denoising autoencoder was implemented in TensorFlow. The encoder is composed of two dense layers. The first layer has 2 × 200 units and utilizes a rectified linear unit (ReLU) activation function. The second layer further compresses the data into an encoding space of dimension 200, with ReLU activation. Symmetric to the encoder, the decoder consists of two dense layers. The first layer expands the encoded data back to 2 × 200 dimensions using ReLU activation. Finally, the second layer reconstructs the data to their original dimension (937) using a sigmoid activation function. The model was compiled using mean squared error (MSE) as the loss function and optimized using the Nesterov-accelerated adaptive moment estimation (Nadam) optimizer. Training was conducted for 128 epochs with a batch size of 32. Both training and testing were performed on noisy data to facilitate the denoising objective. The quality and utility of denoised spectra were subsequently evaluated through effects on performance in quantification models.

### Quantification Models

The quantification of serotonin was primarily evaluated using state-of-the-art neural network models, as shown in Figure 1C. The two model types applied to analyze the spectra were the CNN, which excels at local feature extraction, and the ViT, which better captures global relationships via self-attention. Further, custom SERS-specific layers were evaluated for the CNN. Both the CNN and the ViT models were implemented in TensorFlow and designed to adapt to SERS spectral data. A core CNN architecture comprised of ReLU and hyperbolic tangent (Tanh)-ReLU paired 1-D convolution layers. The core architectures of the quantification models are described in more detail in the Supporting Information. Three variants of CNN were evaluated with varying additional custom layers. These included the base CNN with a linear final activation output layer, a CNN with a modified custom three-parameter logistic (3PL) activation function, and a CNN model with inherent scale-adjusting capabilities through scaling layers.

The scale-adjusting CNN model was developed with two unique scaling layers implemented. These were a multiscale assessing layer and a local scaling layer. Both layers were utilized prior to the half-peak ReLU layer in the core CNN architecture. The multiscale layer captures features from the input *X*, with three layers sized 8, 25, and 50, each with 8 filters. Each convolutional operation is defined as $C_{i}\left(X\right)=W_{i}\times X+b_{i}$ , where × denotes a convolutional operation, *W* the weight, and *b* the bias. To assess the spectra at different scales simultaneously, the output of each convolutional layer is combined by following the convolutional operations along the feature dimension. The local peak scaling layer in turn was developed to scale regions of the spectra, which were assessed not to be relevant to the outcome variable, identified from the reference spectra of the pure compounds in water. The layer applies a set of scaling factors *s*_*j*_ unique to the number of preregistered regions of interest (or noninterest), which are defined by start and end indices $a_{j},b_{j}$ , in the spectra. The scaling operation for each region is expressed as $S_{j}\left(X\right)=X_{aj:bj}⊙s_{j}$ , where ⊙ denotes the element-wise multiplication. The output of the scaling operation is concatenated to reconstruct the spectra with scaled regions. The modified output layer assessed in both custom-layer CNN models utilizes a three-parameter logistic (3PL) activation function. The ViT architecture in turn consisted of an embedding layer with a patch size of 25 matching the CNN architectures’ initial layer, with a hidden size of 64 and a dropout rate of 0.1. Subsequently, the architecture consisted of 6 transformer blocks containing a multihead self-attention layer (6 heads) and a multilayer perceptron. The transformer blocks each employed Gaussian error linear unit (GELU) activation functions. The architectures of the CNN and ViT models are presented in Supplementary Figure 4.

Each model was evaluated in raw, Savitzky–Golay-filtered, and denoised data. These evaluations incorporated unseen spectra as well as repeat spectra, with spectra defined as repeat if separate measurements of a specific sample were used in training either the denoising autoencoder or the quantification models. Repeat spectra were split into training and validation sets with a 90:10 split, and furthermore, measurements taken from an unseen serotonin-free sample (sample F) were included in the validation set. The remaining unseen samples (D and E) were included exclusively in the test set. Final spectra counts for both data sets consisted of 218 training spectra, with a validation set of 46 and a test set of 54 spectra. Hyperparameter tuning and architecture search for both the CNN variants and the ViT were conducted iteratively, guided by the model’s performance on the validation set. Each model variant for both the CNNs and the ViT was trained 100 times, with an ensemble average used for evaluation. Models exceeding 1 μM mean absolute error (MAE) in training were dropped from the ensemble to minimize the inclusion of outlier models. Both model types were optimized using the adaptive moment estimation (Adam) algorithm with a learning rate of 0.001, a batch size of 64, and 256 epochs, and compiled with an MAE loss function. Additional evaluation metrics included the mean squared error (MSE) and mean percentage error (MPE). Early stopping with a patience of 64 was employed to mitigate overfitting, and model checkpoints were saved for epochs that minimized validation loss. Reproducibility was ensured by setting random seeds for TensorFlow, NumPy, and the train-test split. Of the 100 trained models in the ensemble, the model with the lowest MAE in the validation set was selected as the final model, which was assessed in the holdout test set.

### Context Representative Interpretable Model Explanations

The reliability and explainability of the final quantification model were assessed using the context representative interpretable model explanations (CRIME) framework developed in this study for machine learning interpretations of data with expected contextual prediction clusters. The CRIME framework expands on the widely applied local interpretable model-agnostic explanations (LIME) framework^37^ by assessing model explanations through contexts. Contexts can be defined within this framework as prominent and consistent explanation outcomes across a number of prediction instances. While contexts can have numerous explanations as to why they are prominent from other explanation contexts, they can be broadly categorized to stem from differing sources of prediction reasoning, such as confounding factors, outliers, measurement errors, or different predictors of the same outcome variable. The framework is summarized in Algorithm 1.
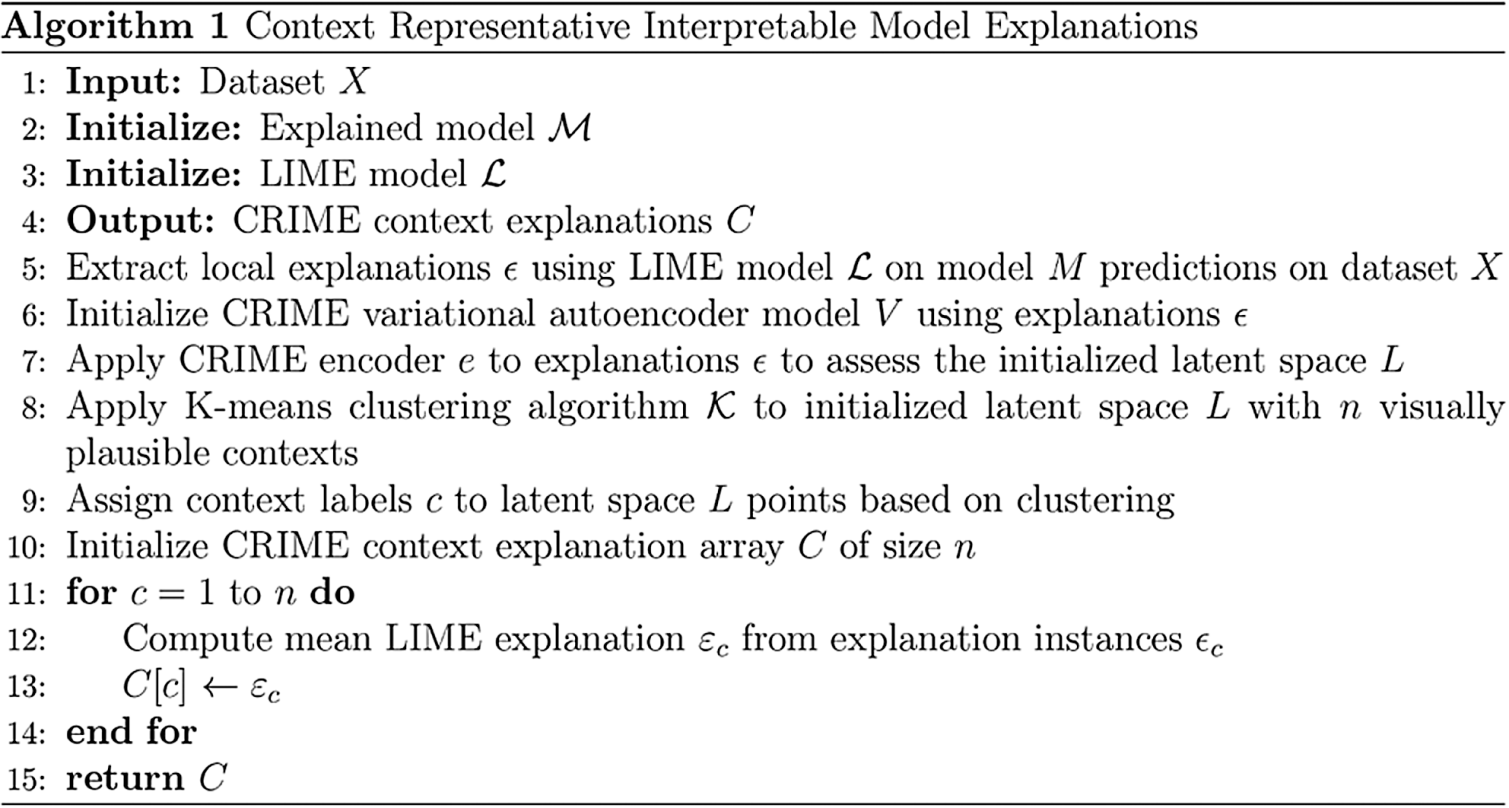


The CRIME framework attempts to identify all prediction contexts of the input data space through the latent space of a variational autoencoder (VAE) trained on the LIME predictions of all instances in the available data. The LIME predictions are flattened with regard to perturbation limits and weights prior to input and are subsequently projected to the two-dimensional latent space. The VAE architecture used in this study consisted of a simple encoder, sampler, and decoder. Notably, the architecture can be fine-tuned depending on the individual requirements of the CRIME framework in future applications. Details regarding the VAE of the CRIME method are described in the Supporting Information. Following training of the VAE, the latent space is utilized to identify context clusters representing all of the possible ways in which the quantification model interprets the input data. The latent space instances are clustered into the final contexts using K-means clustering, and the latent space is visually inspected for selecting the number of clusters. Finally, a mean LIME explanation is assessed by averaging all instances in each cluster to represent the contexts. To identify the defining features of each context representation, normalized LIME feature weights are combined with mean feature values representing the spectral intensities within the context clusters. They are then set in a three-dimensional space, together with normalized feature positions, which are then further clustered into 15 clusters using K-means clustering. Following the clustering of mean spectral feature values, position z-scores, and LIME weights, the clusters are ordered according to the product of their LIME weights and spectral intensities. The five clusters with the highest score are selected to represent the regions of the spectra that contribute most to the contextual predictions.

Following the identification of the most relevant context prediction regions, the highlighted regions of the mean context spectra are assessed against measured clean spectra of the neurotransmitters known to be present in the mixture. To emphasize the explanation weights in the spectra, both the reference clean spectra and the mean context spectra are scaled according to the explanation weights in the specific feature location. To determine the cause or identity of the recognized context clusters, the final mean context indicators are compared to the weighted reference spectra using cosine similarity *S*_cos_, which is defined in eq 1. Cosine similarity is a method that indicates the size-independent directional similarity of two vectors. When comparing two SERS spectra, its use can be interpreted as their direct similarity, with scores of 1 indicating identical spectra, 0 orthogonal spectra, and −1 completely opposite spectra.

1 $$S_{\cos}=\frac{A\cdot B}{∥A∥∥B∥}$$

### Benchmarking

Each segment of the framework presented in this study is carefully benchmarked against alternative models or methods, previously established benchmarks, or common practices in the SERS domain.

The utility of the denoising autoencoder was assessed by measuring performance in the raw and denoised data and, additionally, comparing it with fifth-order polynomial second Savitzky–Golay derivative processing with a window length of 33,^4^ which was previously assessed as a reference standard, therefore allowing for a direct comparison between preprocessing methods. The developed CNN architecture was benchmarked against simpler architectures without Tanh-ReLU pairing layers. Furthermore, the best-performing quantification model was assessed against methods used previously to quantify neurotransmitter concentrations as well as other machine learning methods, including partial least-squares regression (PLSR), random forests, support vector machines (SVM), and extreme gradient boosting (XGBoost). The primary benchmark for serotonin quantification accuracy is the previous study by Kasera *et al*.^4^ Hyperparameter tuning was determined using a grid search and 3-fold cross-validation. The searched parameters are included in the Supporting Information. Final model robustness was tested by using perturbation testing. Gaussian noise was added in two ways across the input spectra: first through applying it across the entire spectra to assess overall noise tolerance, and second through applying noise to identified relevant spectral regions to assess how the model can adapt to noise by assessing contextual cues from neighboring regions. In total, four noise levels were assessed at 5% noise, 10% noise, 20% noise, and 30% noise for both tested methods. Robustness of predictions was assessed using a 0.5 μM MAE cutoff.

For comparison with CRIME, feature importance and model explainability were assessed using logic explained networks (LENs)^38^ and Shapley additive explanations (SHAPs).^39^ The LEN was implemented using PyTorch in Python. In order to apply the LEN, the spectral input data was sectioned into discrete categories, and concept mapping was done through taking the mean of the min-max scaled feature map activations across each layer of the model. Each concept corresponded to approximately 25 *x*-axis points across the SERS spectrum, corresponding to approximately half a peak. Further details of the LEN implementation are described in Supporting Information. SHAP values were in turn calculated using the GradientExplainer tool due to its suitability for neural network models. The SHAP analysis was performed independently for subsets of samples belonging to each of the previously defined discretized outcomes. Mean SHAP values were computed across all spectra in their respective categories and were visualized across an average spectrum in each category.

## Results

### Denoising Autoencoder

In this work, the utility of deep learning methods was assessed for identifying target serotonin concentrations from SERS measurements with CB[8] additives as chemical spacers. To this end, a denoising autoencoder and neural network quantification models were developed using 682 spectral measurements taken from water- and artificial urine-based samples, with concentrations of serotonin ranging from 0 to 9 μM. The denoising autoencoder was implemented to augment the urinary SERS spectra by reintroducing serotonin-based signals from biological noise arising from the urine matrix. The denoising autoencoder was primarily evaluated through its influence on the downstream model predictions, compared to Savitzky–Golay denoised spectra and unprocessed raw spectra. Following training, the denoising autoencoder was able to robustly reconstruct the clean data from noisy inputs in the test set (MSE = 0.025). Examples of input noisy data and subsequently denoised spectra are presented in Supplementary Figure 5A–C. Similar trends are observed in denoising experimental urine measurement spectra, which have been presented in Supplementary Figure 5D–F.

### Quantification Models

Four different neural network models were evaluated in raw as well as Savitzky–Golay and autoencoder-denoised data sets. These included the ViT model, the linear output layer CNN model (CNN_L_), the three-parameter logistic output layer CNN model (CNN_3PL_), and the scale-adjusting three-parameter logistic output layer CNN model (sCNN). For all data sets, all four ensembles and the final selected models showed strong performance in the validation set. The models were then applied on the test set, which was composed of unseen concentration combinations by either the denoising autoencoder or the neural network quantification models. None of the models were able to reach satisfactory differentiation of serotonin from the other neurotransmitters in the raw urine data set (ViT: MAE = 1.17 μM, MPE = 24.46%, CNN_L_: MAE = 0.70 μM, MPE = 22.39%, sCNN: MAE = 0.95 μM, MPE = 26.97%, CNN_3PL_: MAE = 1.14 μM, MPE = 35.34%) or the Savitzky–Golay denoised data set (CNN_L_: MAE = 1.30 μM, MPE = 31.97%, sCNN: MAE = 1.27 μM, MPE = 37.09%, CNN_3PL_: MAE = 1.72 μM, MPE = 55.64%). Notably, none of the 100 trained ViT models were able to converge below the selected 1 μM MAE threshold in training. However, in the denoised data set, all models were capable of robust quantification of serotonin, with the CNN_3PL_ model (MAE = 0.15 μM, MPE = 4.67%) and the sCNN model (MAE = 0.11 μM, MPE = 3.52%) outperforming both the ViT model (MAE = 0.30 μM, MPE = 8.09%) and the CNN_L_ model (MAE = 0.30 μM, MPE = 7.45%). Final model performance on raw and denoised data sets in the validation and test sets for each model is presented in Figure 2. Ensemble model predictions are presented in Supplementary Figure 6. Savitzky–Golay benchmarking results are visualized in Supplementary Figure 7A, B for both ensemble and final model results. Numeric results for all three data types in the validation set are presented in Supplementary Table 3.

**Figure 2:**
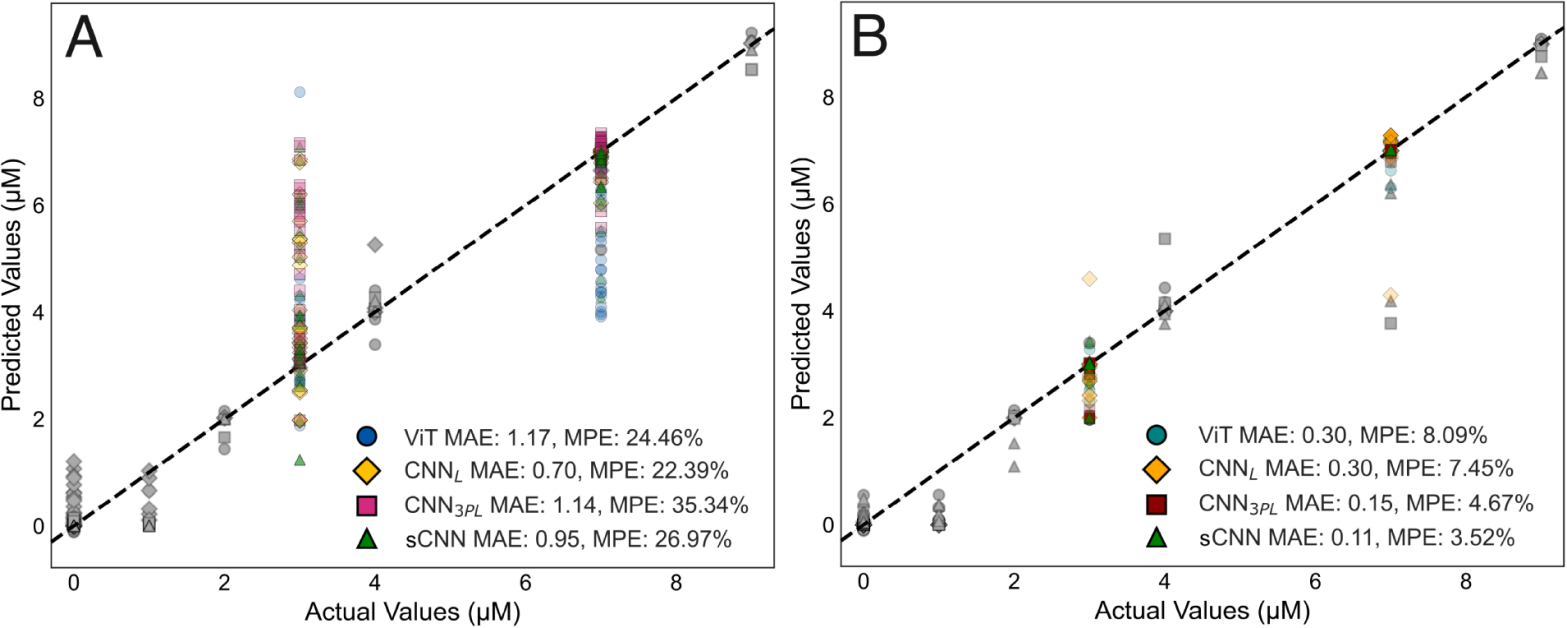
Results of the final models in the validation and test sets for the four model types in both raw (A) and denoised data sets (B). Validation set results are shown in gray, and test set results are shown in color: the linear CNN model is shown in yellow (diamond), the vision transformer model in blue (circle), the scale-adjusting CNN in green (triangle), and the three-parameter logistic output layer CNN model in red (square). A dashed line (*y* = *x*) represents perfect predictions. The values shown were obtained from the final test set. Validation set results are presented in Supporting Information Table 3. MAE = mean absolute error, and MPE = mean percentage error.

### CRIME Framework

The CRIME framework was fit on a VAE by using the LIME explanations of the CNN_3PL_ predictions. This setting was selected due to its strong performance across both the validation and test data. Following the K-means clustering, the latent space of the VAE was clustered into six contexts. The latent space is visualized in Figure 3, depicting the contexts separately as well as the original concentration ranges for each measured spectrum. Among the clusters, four distinct contexts were identified (contexts A, B, C, and F), as well as one intermediate context (context E) and one outlier context (context D). Observably, contexts B and C consisted of samples at the low concentration ranges and serotonin-absent samples, indicating that there is a significant alternative predictive signal present. The mean LIME explanations for each CRIME context cluster are presented in Figure 3A–F. Peak regions were selected further from the most prominent clusters in a three-dimensional representation of the spectral intensities, explanation weights, and position z-scores. The peak-region cluster plots are visualized in Supplementary Figure 8A–F. Contexts A (S_cos_ = 0.87), E (S_cos_ = 0.46), and F (S_cos_ = 0.54) were correctly associated with serotonin, while contexts B and C were associated with dopamine (S_cos_ = 0.98) and epinephrine (S_cos_ = 0.97), respectively. Complete cosine similarity values between mean CRIME context spectra and reference neurotransmitter spectra are presented for each CRIME context in Supplementary Table 4.

**Figure 3:**
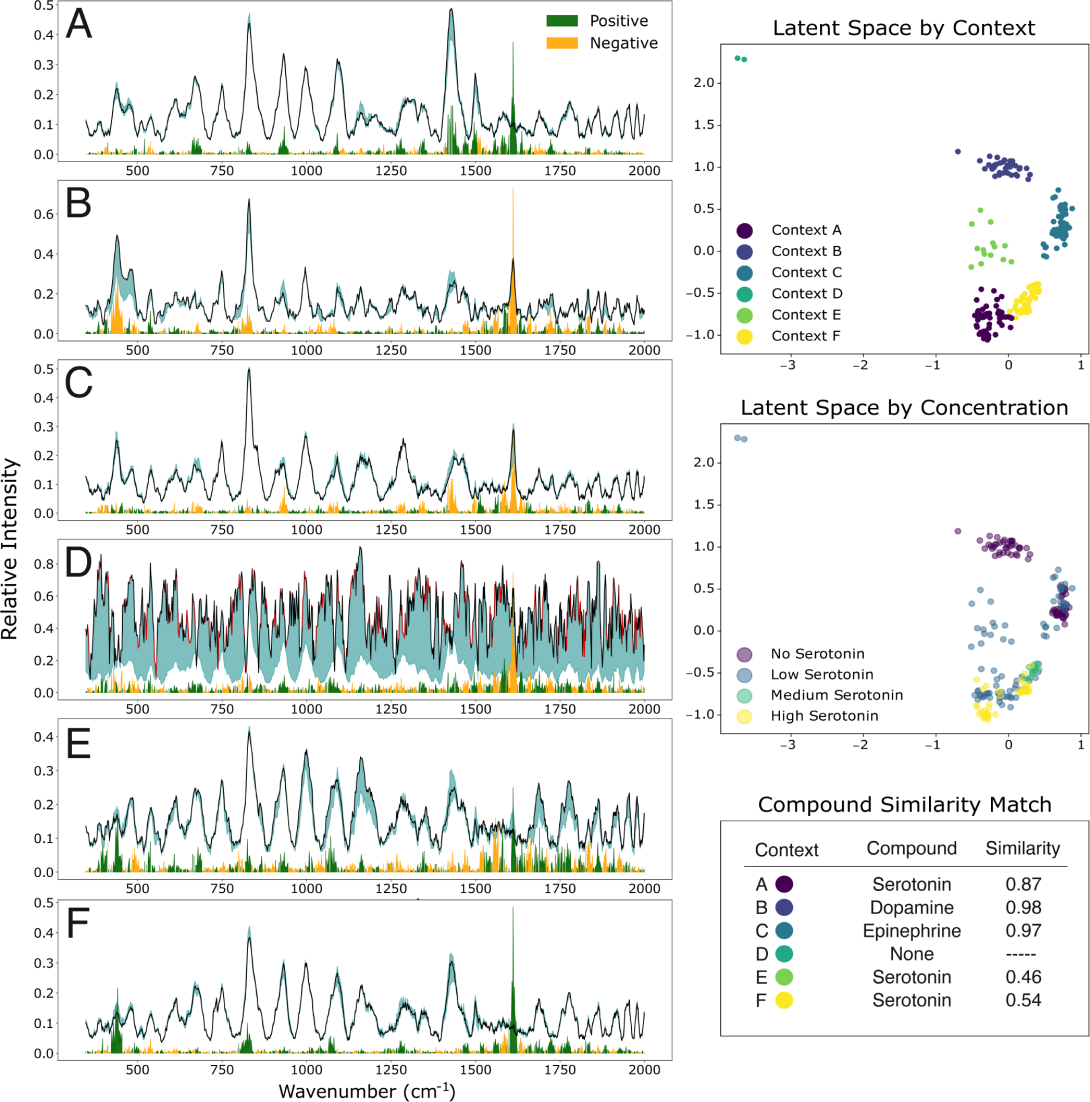
Results for context representative interpretable model explanations (CRIME) analysis. Six distinct contexts were identified, which are visualized across mean spectra in subfigures A–F. Positive prediction weights are presented in green, negative prediction weights in yellow, and perturbation limits have been shaded in teal. Red regions in the mean spectra correspond to average perturbation limits at either the top or bottom of the feature weight range, for the simplicity of the plot. Latent spaces are visualized by context and concentrations, and compound similarity matching was done by using cosine similarity. The highest similarity score is presented alongside that of the matched compound.

### Benchmarking

The neural network models were benchmarked across different architectures and against other machine learning models, with the results summarized in Table 2 and Supplementary Figure 9. The benchmarking results of the CNN architectures are presented in Supplementary Table 2. Of all the benchmarked models, the PLSR model trained with autoencoder-denoised spectra showed the best performance among non-neural network-based models with an MAE of 0.70 μM, a 6-fold error compared to the sCNN. Models trained with denoised spectra near universally showed superior performance when compared to raw spectra-trained models or second derivative Savitzky–Golay denoised spectra-trained models with the exception of the random forests model. Lastly, perturbation testing revealed the final neural network quantification model to be robust to noise, with model predictions crossing the set threshold (MAE < 0.5 μM) at 30% localized noise and 10% universal noise.

**Table 2:** Comparison of Test-Set Mean Absolute Errors Across Different Machine Learning Models for Serotonin Quantification from SERS Spectra^a^

Data Set	XGB (μM)	PLSR (μM)	RF (μM)	SVM (μM)	CNN_3PL_ (μM)	CNN_L_ (μM)	sCNN (μM)	ViT (μM)
Denoising Autoencoder	0.78	0.70	0.93	0.96	0.15*	0.30	0.11*	0.30
Raw Spectra	0.82	2.05	0.88	1.26	1.14	**0.70**	0.95	1.17
Savitzky–Golay	1.15	2.25	1.46	1.37	1.72	1.30	1.27	+
Kasera et al.^4^	-	**0.52**	-	-	-	-	-	-

a Best-performing model MAEs within each data set have been bolded, and the two best models overall have been marked with an asterisk (*). Additionally, for baseline comparison, the mean absolute error of the previously published PLSR model has been presented. sCNN = convolutional neural network with scaling layers and three-parameter logistic (3PL) output layer, CNN3PL = convolutional neural network with 3PL output layer, CNNL = convolutional neural network with linear output layer, SVM = support vector machine, RF = random forests, PLSR = partial least squares regression, XGB = extreme gradient boosting, ViT = vision transformer. + indicates that the ViT model could not converge in training using a 1 MAE cutoff using Savitzky–Golay denoising.

To compare the context explanations to methods of global explainability, the LEN and SHAP frameworks were evaluated as a reference standard. The mean feature activations of the CNN_3PL_ model across all layers are presented in Supplementary Figure 10. The LEN identified logic statements explaining the four categories of serotonin concentrations with fair (0.69, no serotonin) to excellent (0.98, medium serotonin) explanation accuracy. The logic statements are visualized in Supplementary Figures 11–14. Peak regions near wavenumbers of 800, 1000, 1200, and 1450 cm^–1^ were consistently selected within the first-order logic (FOL) statements for all concentration ranges and were deemed to be relevant for serotonin concentration prediction. SHAP values were assessed for all concentration ranges separately and have been visualized on an averaged spectrum in Supplementary Figure 15.

## Discussion

Within the present study, a comprehensive framework of spectral quantification from complex biological media was developed, consisting of data preprocessing and denoising, bioquantification, and model explanation through the CRIME framework. To this end, data from 318 spectra from lyophilized urine media, as well as 364 spectra from water media, were utilized for the development of neural network models for denoising of urine backgrounds and quantification of serotonin. The trained denoising autoencoder improved prediction outcomes near-universally across all model types and enabled robust quantification compared with the raw and Savitzky–Golay-processed spectra. The assessed state-of-the-art neural network models substantially outperformed traditional machine learning methods commonly used in the SERS domain, with the CNN_3PL_ and the scale-adjusting sCNN models yielding the lowest prediction errors. Notably, the models developed in the present study substantially outperformed existing methodologies.^4^ Additionally, the developed CRIME explainability framework identified the spectral contexts in which the model was reliably assessing the relevant serotonin peaks, as well as contexts representing confounding factors or other sample artifacts, which were not readily observable from the outputs of the LEN or the SHAP explanations.

It can be assessed that the custom layers developed in this study for the sCNN and CNN_3PL_ models significantly improved quantification performance. The final output layer of a model acts as a type of calibration curve to the final transformations from the input data. In this sense, a regression task of biomolecular quantification is effectively a calibration task. The use of logistic calibration curves can often yield a better fit on data compared to linear curves, as near the limit of quantification an assay can become saturated, or as the values approach the limit of detection, the linearity of the signal can deteriorate. The scaling layers similarly were able to improve the predictions, most likely due to their added capability to handle variation in scale. This was surprisingly relevant despite the present samples all being measured using an identical setup, spectrometer, chemicals, and location, which would suggest limited reasons for significant changes in intensity scaling. These layers should be further assessed in different SERS tasks to confirm their utility in subsequent studies.

The CRIME framework within this study was able to effectively explain the CNN model, exhibiting a significant improvement in the understanding of the model decisions. The found contexts were associated with the neurotransmitters present in the mixture, and it could be assessed that the two largest contexts were accurately representing serotonin, while two contexts were associated with unwanted signals, one context was ambiguous in its associations, and last, an outlier context was observed. Regarding the outlier context, it can be attributed to a number of possible measurement errors resulting in null spectra. Laser malfunctions, improper sample loading, lack of aggregation, or other technical factors could result in a high-noise, low-signal spectrum, which, following scaling, would resemble the observed pattern. The dopamine- and epinephrine-associated contexts reveal the imbalances present within the data set. The misidentified contexts were revealed to consist exclusively of samples with low or absent serotonin concentrations. Following this observation, it can be concluded that within the present data set, a correlation existed between a lack of serotonin and the presence of other neurotransmitters. Therefore, expanding the data set to include samples with low concentrations of all neurotransmitters could remedy the apparent inability of the model to generalize to lower serotonin concentrations. To illustrate this point, a small data-augmentation-based supplementary analysis is presented in the Supporting Information which shows that the CNN model substantially improves its low-range serotonin prediction in the validation set. When compared directly to the explanations provided by the trained LEN and SHAP explainers, it can be seen that there is a robust association of peaks with serotonin in all three methods. However, SHAP explanations could not effectively communicate the potential presence of confounding factors. Similarly, LEN statements, while effective in identifying potential confounders, are complex enough to make their presentation unintuitive. However, this could be remedied through a similar context-seeking variation of LEN. Within applications where the identification of all prediction reasoning is crucial, the application of the CRIME framework could see benefit over current best practices for global explainability.

It must be highlighted that the CRIME framework combined with SERS could see clinically relevant use through acting as the first step in biomarker discovery trials. Instead of assessing individual biomarkers of disease through established hypotheses, a biomarker discovery study could be initiated in a nontargeted, hypothesis-generating fashion. Applying a machine learning model on raw spectra presently is not advisable due to the lack of confidence in the model assessing true biomarkers as opposed to confounding factors. The exact identification of the signaling biomarkers is challenging when global explainability methods are used for peak detection, as the spectral signals could be a result of multiple overlapping compounds. However, were the CRIME framework applied, individual target biomarkers could be identified through contexts uniquely and subsequently assigned to the likely biomarkers through a complete library of present compounds, as well as hypothesized biomarkers. With the advent of computational hypothesis-generating methodologies such as Mendelian randomization,^40^ future biomarker discovery trials could see a significant change in early-stage methodology and approach. Similarly, the effects of potential comorbidities or medication on spectral signals could be identified through the association of contexts to such groupings, and therefore, their effects could be mitigated within a biomarker analysis. For example, in a diagnostic task predicting schizophrenia, there would be a significant risk of misidentifying the effects of comorbidities and antipsychotic medications as disease biomarkers. However, following a CRIME analysis, different contexts could be identified corresponding to these effects or their known biomarkers. Various strategies could then be applied to remove these effects from the spectra. Notably, the robust performance of the CRIME framework in the data set used in the present study is highly promising, as its modest size, inherent bias, and high levels of confounding closely resemble realistic data constraints in most clinical data sets. To validate the CRIME framework’s explainability and clinical utility, ongoing efforts are being directed toward a biomarker study, which aims to establish the framework’s effectiveness in such relevant clinical scenarios.

There are several limitations to consider in this study. The development of a denoising autoencoder in patient urine samples as opposed to artificial urine samples could prove to be more challenging. While it could be explained by the CRIME framework, the neural network models were not always able to assess the association of the peaks to the target serotonin compound directly and instead assessed the presence of dopamine or epinephrine as a predictor of the absence of serotonin. The CRIME framework in turn presents limitations inherent in LIME explanations, as the explanations are dependent on a simpler model fit to complex model predictions and, as such, do not completely represent an explanation of the actual model. Additionally, it could prove challenging to identify the true reasoning behind CRIME contexts if there were more potential confounding compounds or effects present. Similarly, while in the present task it was feasible to calculate the LIME explanations for all instances, this might not be scalable to larger data sets. Finally, although the inherent structure of SERS spectra effectively reduces data dimensionality, the data set size in this study remains modest. Further validation is therefore necessary on larger and more diverse data sets to fully assess the generalizability of the developed models, especially in more complex tasks such as multiplexing.

In conclusion, the present study set out to develop an interpretable deep learning framework that was capable of achieving three main aims: to perform enhanced single analyte detection from a high-variance, confounder-rich biomarker matrix in urine; to recognize and adjust for method-based variation in SERS measurements; and last, to identify all confounders that interfered with the correct prediction logic of the quantification model. A denoising autoencoder was developed to improve the targeting of relevant neurotransmitter peaks. To assess serotonin quantification in raw and denoised spectra, three different state-of-the-art neural network models were developed: a CNN with a three-parameter logistic output layer, a scale-adjusting CNN, and a ViT model. In addition, all models were compared with other machine learning methods. Finally, a novel model explainability framework, CRIME, was built around LIME explanations through the assessment of prediction contexts by using a combination of VAE and clustering algorithms. The model interpretability was compared between the novel framework and global prediction methods: LEN and SHAP. Within this study, it was shown that the denoising autoencoder substantially improved the predictive capabilities of applied machine learning models, of which the developed three-parameter logistic output layer CNN outperformed other models assessed. Moreover, model explainability was strongly enhanced by the CRIME framework. To our knowledge, this marks the first instance where an autoencoder has been successfully applied to biological “noise” within the SERS domain. Within the chemical spectral domain, the CRIME framework promises to enable the deployment of spectral quantification methods to directly identify disease features in biological fluids, which could be further refined into specific biomarkers through the identification of relevant contexts.

## Supplementary Material



## Data Availability

The developed code for the CRIME framework can be found in the following GitHub repository: https://github.com/jkz22/CRIME

## Notes

Supplementary sections for experimental methods, neural network architectures, AI explainability, benchmark hyperparameter search, and retrospective model development. Supplementary figures for pure compound spectra, neural network architectures, denoising autoencoder performance, validation set ensemble predictions, CRIME context relevance cluster visualization, quantification model benchmarking, feature activation maps for the final CNN model, visualized LEN explanations, and visualized SHAP explanations (PDF)
