# Supplementary material for: Explainable Deep Learning Framework for SERS Bioquantification
Source: ACS Sens. 2025 Sep 2;10(9):6597–606. doi: 10.1021/acssensors.5c01058 (PMC12481578; doi:10.1021/acssensors.5c01058)
Supplement: Supplementary file 1 [file se5c01058_si_001.pdf]

Supporting Information: Explainable Deep Learning Framework for SERS Bio-quantification

Jihan K. Zaki,[†] Jakub Tomasik,[‡] Jade A. McCune,[†] Sabine Bahn,[‡] Pietro Liò,[*,¶] and Oren A. Scherman[*,†]

†*Melville Laboratory for Polymer Synthesis, Yusuf Hamied Department of Chemistry, University of Cambridge, Lensfield Rd, CB2 1EW, UK*

‡*Department of Chemical Engineering and Biotechnology, University of Cambridge, Philippa Fawcett Drive, CB3 0AS, UK*

¶*Department of Computer Science and Technology, University of Cambridge, 15 JJ Thomson Ave, CB3 0FD, UK*

E-mail: pl219@cam.ac.uk; oas23@cam.ac.uk

Experimental Methods

All initial reagents were sourced from Alfa Aesar and Merck and were utilized in their received state unless otherwise specified. Cucurbit[8]uril was prepared following established literature protocols.[1,2] Millipore water with a resistivity of 18 M Ω·cm was employed in all experiments, unless otherwise indicated. Fresh standard stock solutions of neurotransmitters, specifically dopamine, epinephrine, and serotonin, were prepared at varying concentrations to simulate potential interfering background analytes. Gold nanoparticles (AuNP) with a diameter of 60 nm stabilized by citrate were procured from British Biocell International (BBI). Lyophilized urine samples designated as Calibrator Lot No. 150 and Control Level II Lot No. 230 were obtained from RECIPE ClinChek-Control and were reconstituted in dilute hydrochloric acid as per the supplier's guidelines.

Spectra for both Raman and SERS were collected with a 785 nm laser operating at 17.5 mW, using an Ocean Optics QE65000 Spectrometer. Each spectrum was acquired over a 10 s interval. AuNPs with a 60 nm diameter were first centrifuged at 12,000 rpm for 45 s, repeated twice, and 900 µL of the supernatant were removed. Subsequently, a sample preparation sequence was followed: neurotransmitters (dopamine, epinephrine, and serotonin) were first added, followed by 50 µL of the centrifuged AuNPs, then 20 µL of CB[8] at a final concentration of 20 µM, and finally, 50 µL of thawed urine, which had been initially stored on ice. An identical procedure was replicated, replacing urine with water for control experiments.

Supplementary Table 1: **Band assignment of serotonin SERS peaks.** Vibrational assignments and reference peaks are from Borah and Devi[3]. Peaks were matched to the closest experimental reference when available. Bands assigned only by theory are marked with an asterisk (*). ν = stretching, β = in-plane bending, τ = torsion.

Identified peak (cm^{-1})	Ref. Raman (cm^{-1})	Vibrational assignment
670	678	$\beta(C_2-C_3-C_4), \beta(C_3-C_4-C_5), \beta(C_4-C_5-C_6), \beta(C_3-C_2-N_{16}), \beta(C_2-N_{16}-C_8)^{25}$
932	929*	$\nu(C_{20}-C_{17})^{39}, \beta(H_{24}-N_{23}-C_{20})^{18}$
996	998*	$\nu(C_7-C_{17})^{10}, \nu(C_{20}-N_{23})^{19}, \tau(H_{21}-C_{20}-C_{17}-C_7)^{10}$
1106	1104	$\beta(H_{24}-N_{23}-C_{20})^{22}, \beta(H_{18}-C_{17}-C_{20}), \beta(H_{21}-C_{20}-N_{23})^{39}$
1206	1200*	$\nu(C_5-O)^{10}, \beta(H_{16}-C_6-C_1)^{28}$
1317	1314	$\beta(H_{24}-N_{23}-C_{20})^{17}, \beta(H_{18}-C_{17}-C_{20}), \beta(H_{21}-C_{20}-N_{23})^{12}, \tau(H_{19}-C_{17}-C_8-C_7)^{15}$
1424	1432*	$\beta(C_{17}H_2)^{39}$
1499	1498	ν(C=C)^{43} (ring)

Neural network architectures

Quantification Models

Both the CNNs and the ViT models were implemented in TensorFlow and designed to adapt to SERS spectral data. The CNN architecture comprised sequential layers optimized for 1D convolution operations, and the core CNN architecture was used in all trained CNN models, where the initial layer is a convolutional layer featuring 8 filters and a large kernel size approximately the width of half a peak (25 datapoints), aimed to capture broader features in the spectrum. This initial layer employs a Rectified Linear Unit (ReLU) activation function and reduces sequence length through striding. Intermediate layers employ paired hyperbolic tangent (Tanh) and ReLU activation functions, designed to capture complex patterns while maintaining non-linearity. The combination of consequential Tanh and ReLu layers is to direct the model to assess the upper half of the identified general peaks from the sweeper layer. These layers maintain the same padding to avoid changes in sequence length. The model contains two Tanh-ReLu paired layers with 2×16 and 2×32 filters respectively, with

a filter size of 9. The final convolutional stage employs 64 filters with a smaller kernel size of 2 using ReLU activation, aimed to capture fine-grained details in the data. Subsequently, the data is flattened and passed through two fully connected layers employing the same Tanh to ReLu structure with 32 and 16 nodes respectively, to serve the regression task. The core architecture of the CNN models was benchmarked against similar architectures with Tanh-ReLu pairs replaced with ReLu pairs, inversed ReLu-Tanh pairs, or single ReLu layers. Each of the benchmark architectures were trained and validated as described in the methods section, and the results are summarized in **Supplementary Table 2**.

Supplementary Table 2: **Comparison of core CNN architectures.** All architectures were trained on denoised urine medium datasets, and validated using the holdout test set. ReLu = Rectified linear unit, Tanh = Hyperbolic tangent, MAE = Mean absolute error, MPE = Mean percentage error.

**Error**	**Tanh-ReLu**	**ReLu-Tanh**	**2xReLu**	**ReLu**
MAE (µM)	0.30	0.56	0.88	0.78
MPE (%)	7.45	17.68	20.71	16.61

Supplementary Table 3: **Validation set results for neural network models.** sCNN = convolutional neural network with scaling layers and three parameter logistic (3PL) output layer, CNN_{3PL} = convolutional neural network with 3PL output layer, CNN_L = convolutional neural network with linear output layer, ViT = vision transformer.

**Dataset**	**CNN_{3PL}** (µM)	**CNN_L** (µM)	**sCNN** (µM)	**ViT** (µM)
Denoising Autoencoder	0.24	0.13	0.33	0.20
Raw Spectra	0.19	0.31	0.14	0.25
Savitzky Golay	0.30	0.17	0.25	–

AI explainability

CRIME variational autoencoder

Within the encoder of the CRIME VAE, the input data X, is transformed into the mean (μ) and logarithm of the variance ($\log(\sigma^2)$) in a proposed Gaussian distribution in the latent space through a fully connected ReLu layer with 256 nodes. During training, the outputs

of the encoder are passed to a sampling layer which generates a random noise variable ϵ generated from a standard gaussian distribution, which is then transformed using the encoder outputs to draw samples z as such: $z = \mu + \sigma * \epsilon$. The decoder then applies a mirrored dense layer network to the encoder with a ReLu layer with 256 nodes, and a final output sigmoid layer with 3×842 nodes. The model was trained for 128 epochs and a batch size of 32, with the Adam optimizer with a learning rate of 0.001 and using the sum of the mean squared error, and Kullback–Leibler divergence as the loss function.

Supplementary Table 4: **Cosine similarity values across explanation weighted reference spectra and explanation weighted mean context spectra.** Highest similarity values within a context cluster are bolded. X = context.

Reference compound	A	B	C	D	E	F
Serotonin	**0.87**	0.85	0.60	-0.79	**0.46**	**0.54**
Dopamine	0.05	**0.98**	0.91	-0.80	0.29	0.06
Epinephrine	0.0	0.81	**0.97**	-0.79	0.12	0.08

Logic explained network architecture

The LEN architecture was modified to be similar to the original model architecture, consisting of an entropy layer with 164 input nodes, a leaky ReLu layer with 32 nodes, a Tanh layer with 16 nodes, a ReLu layer with 4 nodes, and a final linear output layer. The LEN was trained using weight decay Adam as the optimizer, using binary cross entropy with logits loss as the loss function with a scaled auxiliary entropy loss at a 0.000001 multiplier. The model was trained with 5001 epochs using a learning rate of 0.0001.

Benchmark hyperparameter search

The grids used for the hyperparameter search of the benchmark machine learning models are presented below with the final hyperparameters for the denoised models in bold.

XGBoost

Hyperparameter	Values
colsample_bytree	**0.5**, 0.7, 0.8
learning_rate	**0.01**, 0.1, 0.2, 0.3
max_depth	3, **6**, 9, 12
alpha	1, **3**, 5
n_estimators	100, 300, 600, 900, **1200**

Random Forests

Hyperparameter	Values
max_depth	1, 2, 3, 6, 7, **8**, 10
n_estimators	100, 300, 600, 900, **1200**

PLSR

Hyperparameter	Values
n_components	5, 8, **12**

SVM

Hyperparameter	Values
C	0.1, 1, 10, **50**, 100
epsilon	**0.01**, 0.1, 1
gamma	**scale**, auto

Retrospective model development following CRIME analysis

The CRIME analysis conducted in the present study provided critical insights into the decision-making process of the CNN3PL model, highlighting specific characteristics of the data set that influenced its predictions, particularly at low serotonin concentrations. Two primary issues were identified: (1) a paucity of training examples featuring zero serotonin concentration co-occurring with varied concentrations of other neurotransmitters, and (2) the dataset containing only one category of spectra with a 1 µM serotonin concentration. Targeted data augmentations can be designed to address these limitations, demonstrating the potential to enhance model robustness and extend its predictive capabilities, guided directly by the issues flagged by CRIME.

Two forms of data augmentation were applied to the existing data for spectra in two samples, sample U (representing a blank urine measurement), and sample J (representing urine samples with high epinephrine and dopamine at a 1 µM serotonin concentration). To increase the weight of null samples in the training data, a simple augmentation was applied. Blank urine samples were randomly paired with other spectra in the sample, and were averaged into a single new spectra, effectively increasing the available null sample data by 50%. Subsequently, for the 1 µM serotonin samples, the CRIME analysis indicated that the model might be leveraging the absence of strong dopamine and epinephrine signals rather than directly detecting serotonin. To mitigate this, a targeted augmentation was performed by minimizing key peaks in the 1500-1800 cm^{-1} spectral region. These peaks are absent in serotonin (**Supplementary Figure 1**), and can be predominantly attributed to dopamine and epinephrine (**Supplementary Figures 2 and 3**). The region intensities were replaced with points from low-dopamine and low-epinephrine samples for the erased area. A visualization of the augmentation is presented in **Supplementary Figure 16**.

A CNN3PL ensemble was trained using the exact experimental setup as detailed in the methods section for the initial model training phase. The CNN3PL ensemble following data-augmentations showed significant improvement in distinguishing sample concentrations

in the range of 0 to 1 µM in the validation set (MAE = 0.08, MPE = 3.44%). The test set performance of the CNN3PL ensemble increased modestly after augmentation (MAE = 0.56 µM, MPE = 16.76%), compared with the original model (MAE = 0.15 µM). This can be attributed to inconsistencies introduced by the peak-masking augmentation, which removed only non-overlapping dopamine/epinephrine bands and therefore most likely weakened the model's ability to exploit the remaining serotonin-inclusive peaks. The validation and test set predictions are visualized in **Supplementary Figure 17**.

The improvement in the predictions following augmentations signals that the loss in performance at the lower concentration bounds was primarily a result of the data imbalances inherent in the dataset, and not a result of limitations in the SERS measurements, or the models developed. However, these augmentation results must be interpreted within context. The data augmentations for zero serotonin predictions can be verified to improve the model due to the presence of an unseen sample in the validation set (Table 1, sample F). However, it is impossible to definitively confirm the same for 1 µM predictions, due to a lack of completely unseen samples with a true serotonin concentration of 1 µM. Furthermore, the model trained with this augmented data should not be considered the final, optimized model. Instead, this analysis only serves as a demonstration to illustrate the utility of CRIME. In practical scenarios, the dataset should be substantiated with true measured spectra, which would improve the model without the drawbacks introduced by augmentations.

References

(1) Kim, J.; Jung, I.-S.; Kim, S.-Y.; Lee, E.; Kang, J.-K.; Sakamoto, S.; Yamaguchi, K.; Kim, K. New Cucurbituril Homologues: Syntheses, Isolation, Characterization, and X-ray Crystal Structures of Cucurbit[n]uril (n = 5, 7, and 8). *J. Am. Chem. Soc.* **2000**, *122*, 540–541.

(2) Day, A.; Arnold, A. P.; Blanch, R. J.; Snushall, B. Controlling Factors in the Synthesis of Cucurbituril and Its Homologues. *J. Org. Chem.* **2001**, *66*, 8094–8100.

(3) Borah, M. M.; Devi, T. G. Vibrational study and Natural Bond Orbital analysis of serotonin in monomer and dimer states by density functional theory. *Journal of Molecular Structure* **2018**, *1161*, 464–476.

Supplementary Figures

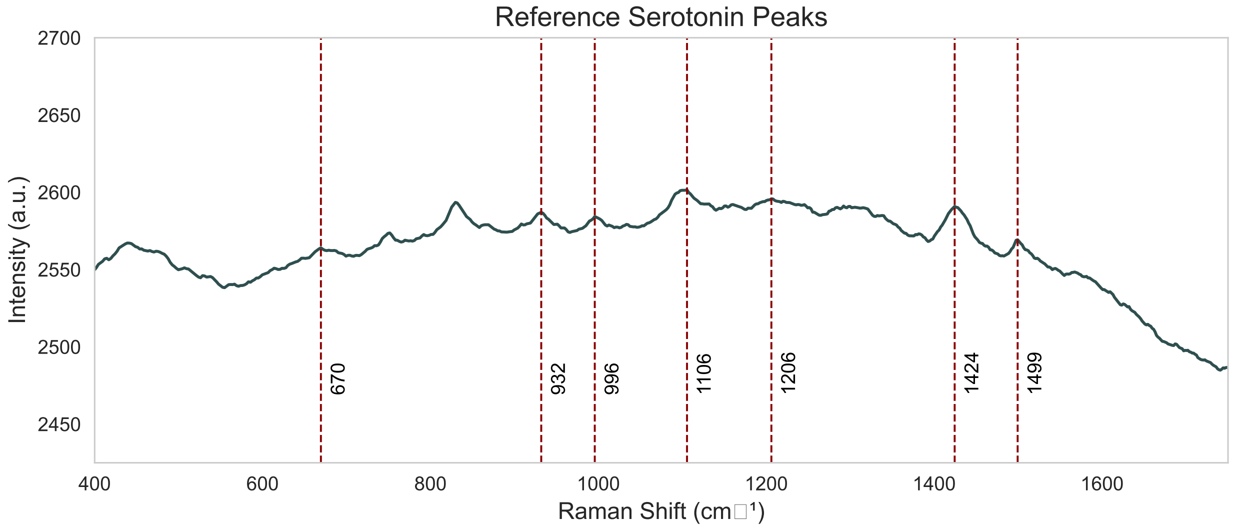

Supplementary Figure 1: **SERS spectra of pure serotonin measured in water.** Serotonin was measured at a 2 µM concentration, and serotonin specific peaks are highlighted using a red dashed line together with specific wavenumbers. Peaks near 439, 749, and 830 were excluded as known Cucurbit[n]uril peaks. Band assignments according to literature are presented in **Supplementary Table 1.**

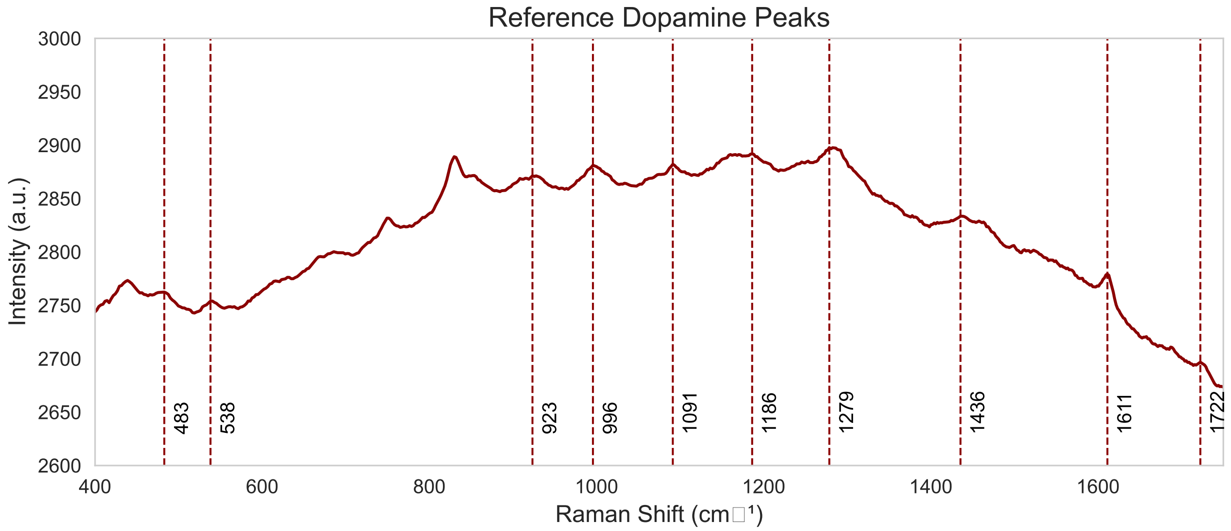

Supplementary Figure 2: **SERS spectra of pure dopamine measured in water.** Dopamine was measured at a 2 µM concentration, and dopamine specific peaks are highlighted using a red dashed line together with specific wavenumbers. Peaks near 439, 749, and 830 were excluded as known Cucurbit[n]uril peaks.

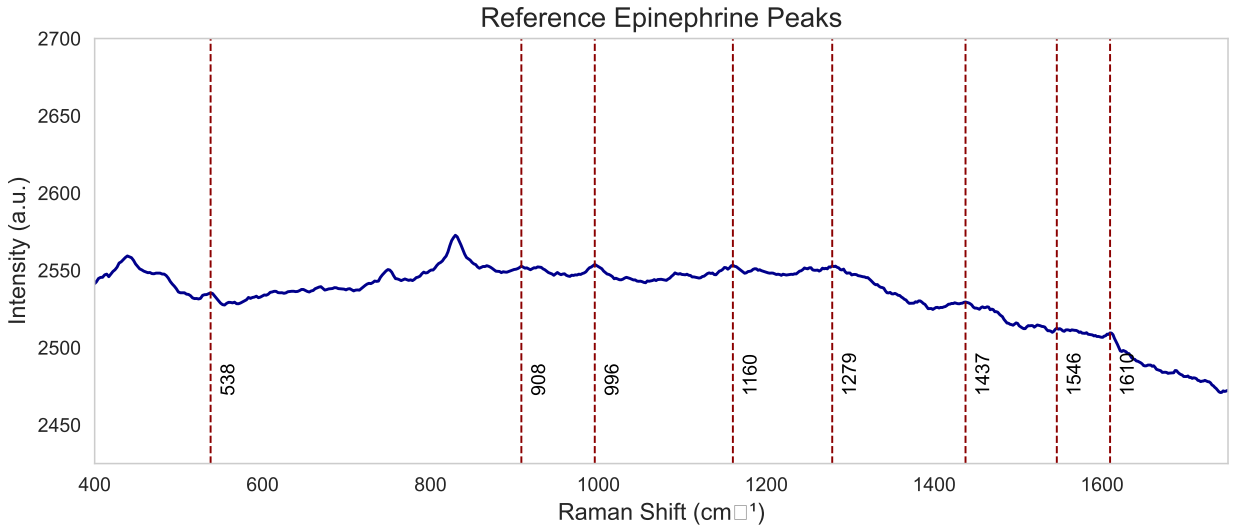

Supplementary Figure 3: **SERS spectra of pure epinephrine measured in water.** Epinephrine was measured at a 2 µM concentration, and epinephrine specific peaks are highlighted using a red dashed line together with specific wavenumbers. Peaks near 439, 749, and 830 were excluded as known Cucurbit[n]uril peaks.

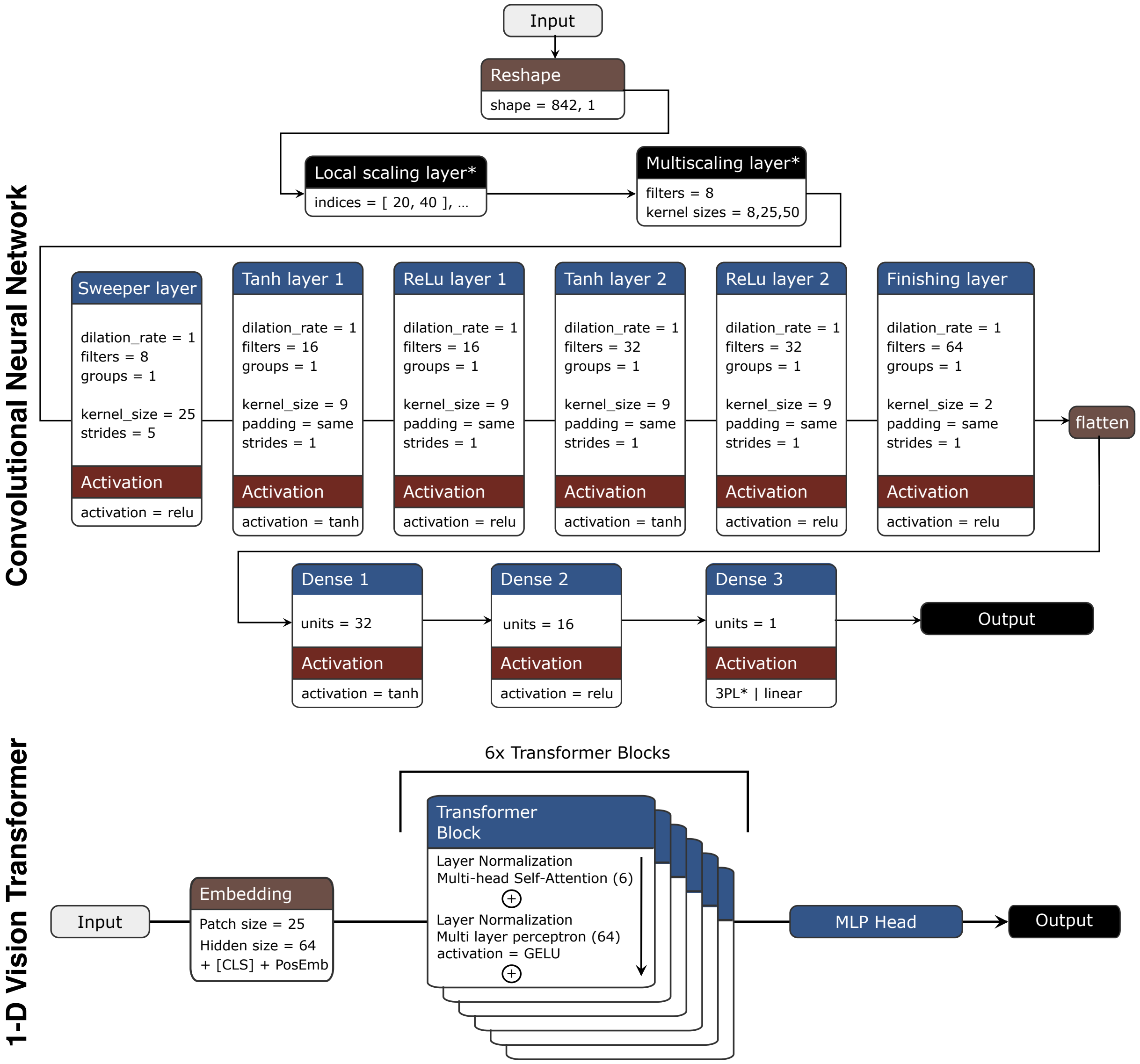

Supplementary Figure 4: **Neural Network Architectures for all Convolutional Neural Network (CNN) variants and the 1-D Vision Transformer (ViT) model.** All CNNs share a core architecture, comprising an initial Conv1D layer (8 filters, kernel size 25, ReLU) followed by two pairs of Tanh-ReLU activated Conv1D layers (16 then 32 filters, kernel size 9), a final Conv1D layer (64 filters, kernel size 2, ReLU), a Flatten layer, and two Tanh-ReLU activated Dense layers (32 then 16 nodes). The layers specific to the sCNN and CNN3PL models are highlighted with an asterisk (*). Within the ViT model, input spectra are divided into patches (size 25) and linearly embedded into 64 dimensions. Positional embeddings are added, and the sequence is processed by 6 Transformer blocks. Each block contains Multi-Head Self-Attention (6 heads) and a Multi-Layer Perceptron (64 dimensions) with GELU activation. Dropout (0.1) is applied. A final MLP head performs the regression task.

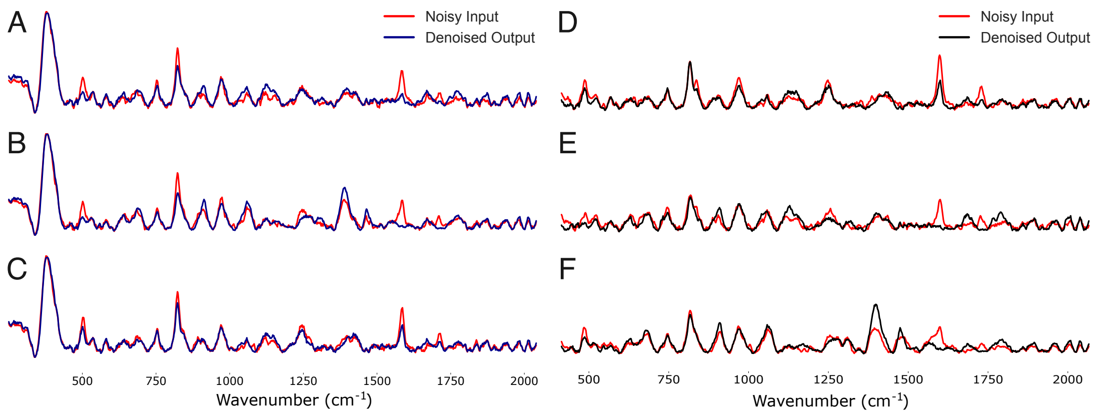

Supplementary Figure 5: **Examples of denoised spectra in the artificial training data (A-C), and in lyophilized urine spectra (D-F).** Y-axis (relative intensity) is omitted for clarity.

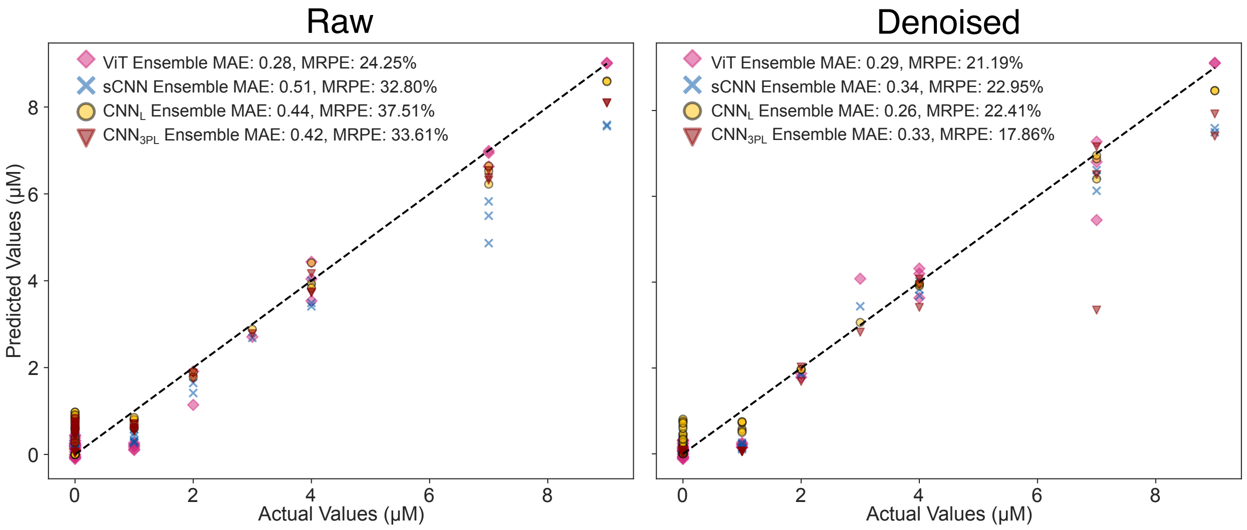

Supplementary Figure 6: **Predictions of the trained ensembles on the validation set for all types of neural networks on both raw spectra and denoised spectra.**

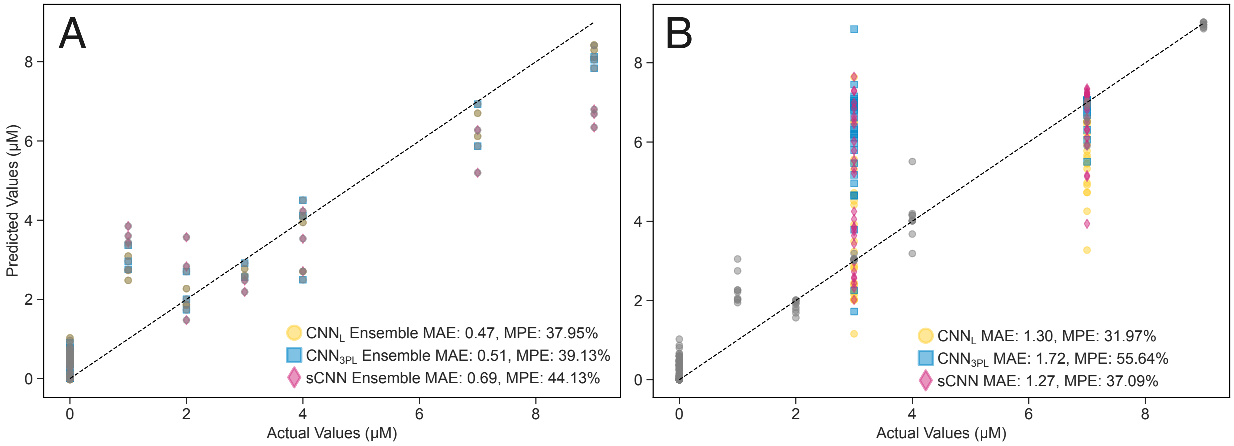

Supplementary Figure 7: **Results of the Savitzky-Golay denoised benchmarks for ensemble (A) and final models (B) for the three neural network model types.** Validation set results were assessed for the ensemble and final models, and are shown in grey, and test set results are shown in color for the final models. The linear CNN model is shown in yellow (circle), the scale-adjusting CNN in pink (diamond), and the three-parameter logistic output layer CNN model in blue (square). The shown values (B) were obtained from the final test set. Validation set results are presented in Supplementary Table 3. MAE = mean absolute error, MPE = mean percentage error.

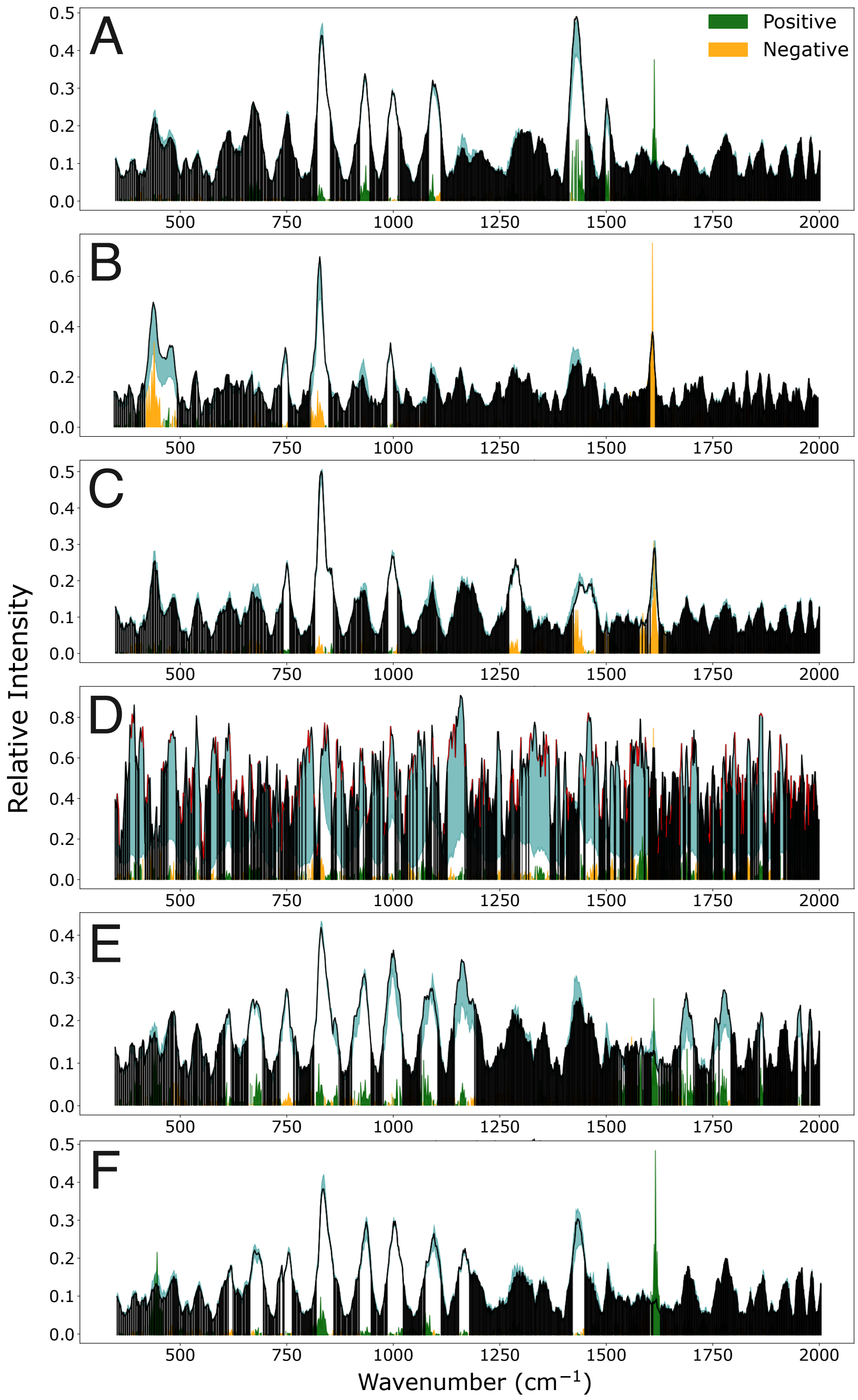

Supplementary Figure 8: **Peak-region clusters of high relevance extracted from CRIME contexts for compound identification.** Context labels correspond to labels in Figure 3. Positive prediction weights are presented in green, negative prediction weights in yellow, and perturbation limits have been shaded in teal. Red regions in the mean spectra correspond to average perturbation limits at either the top or bottom of the feature weight range for the simplicity of the plot. Areas not relevant are marked in black. High-relevance clusters were obtained with K-means clustering of the product of peak height and LIME weights, with the top 5 largest clusters selected.

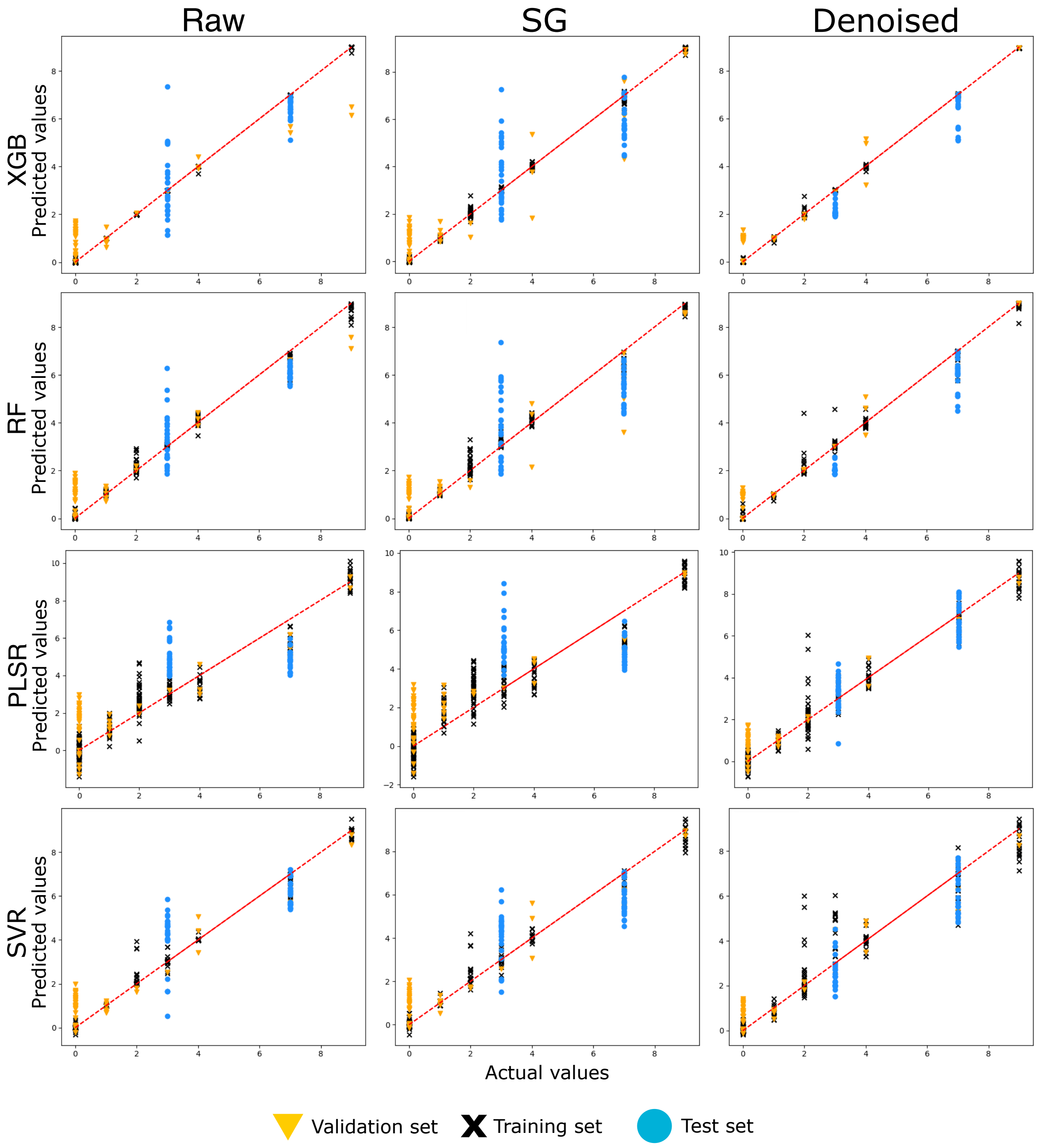

Supplementary Figure 9: **Results for quantification model benchmarking.** Training set predictions are marked in black (cross), validation set predictions in orange (triangle), and test set predictions in blue (circle). XGB = extreme gradient boosting, RF = random forests, PLSR = partial least squares regression, SVM = support vector machine regression, SG = Savitzky-Golay filter.

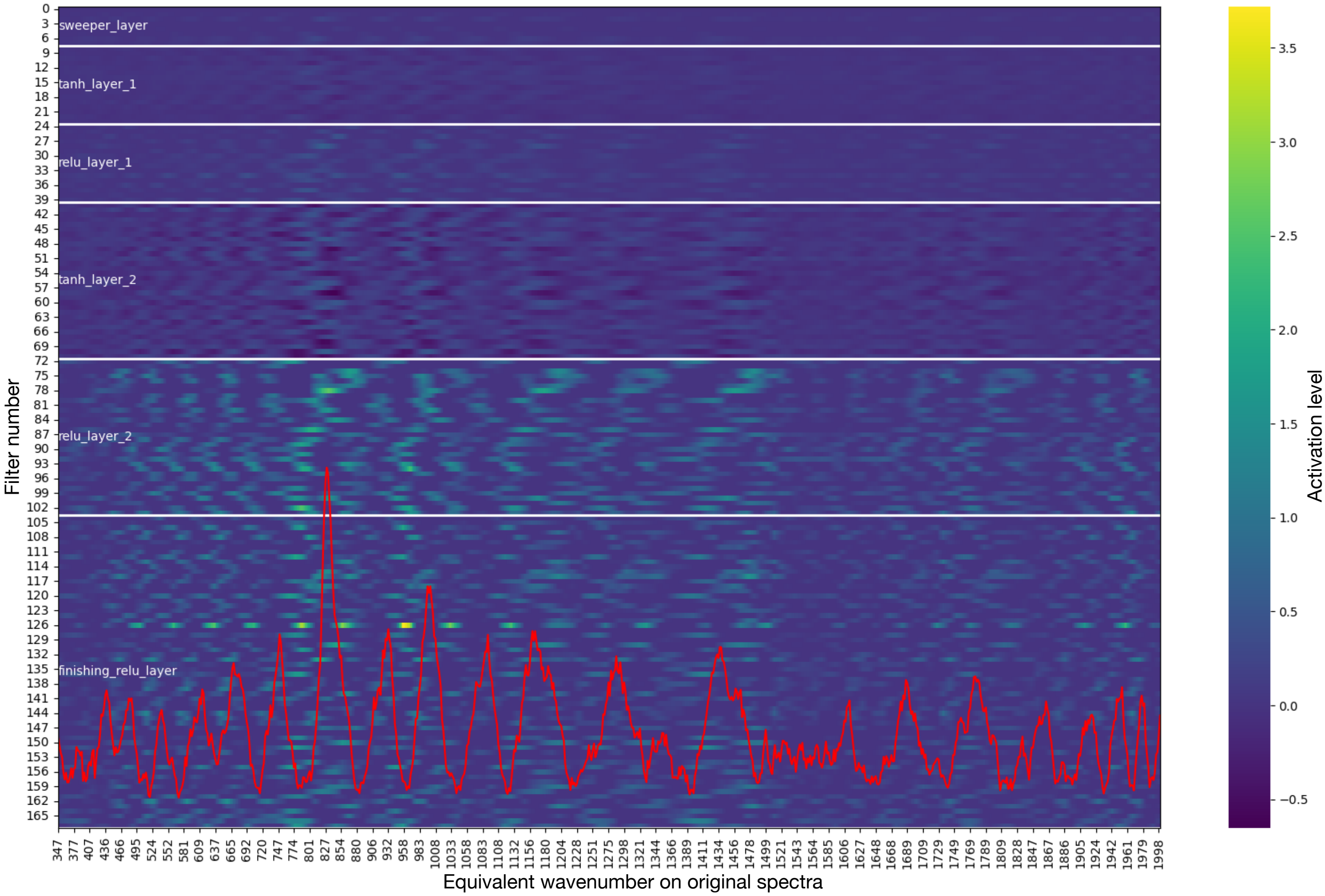

Supplementary Figure 10: **Feature activation map for each convolutional layer in the CNN model overlayed with an example spectra.** SERS spectra is shown in red, and higher activations are marked with a yellow hue, with lower activations marked in blue.

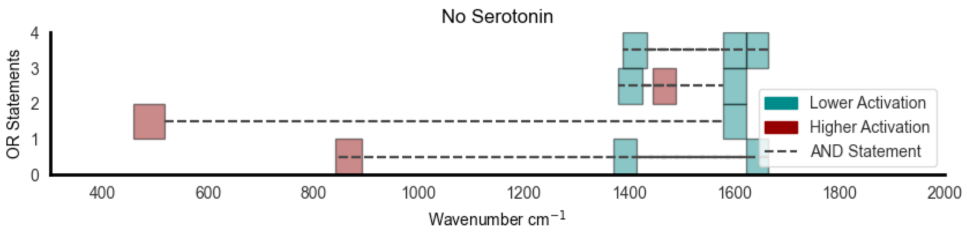

Supplementary Figure 11: **LEN results visualized for samples with no serotonin concentration.** OR statements are separated vertically, and AND statements are presented level with a dashed line connecting the statements. Blue squares denote lower activation and red squares higher activation.

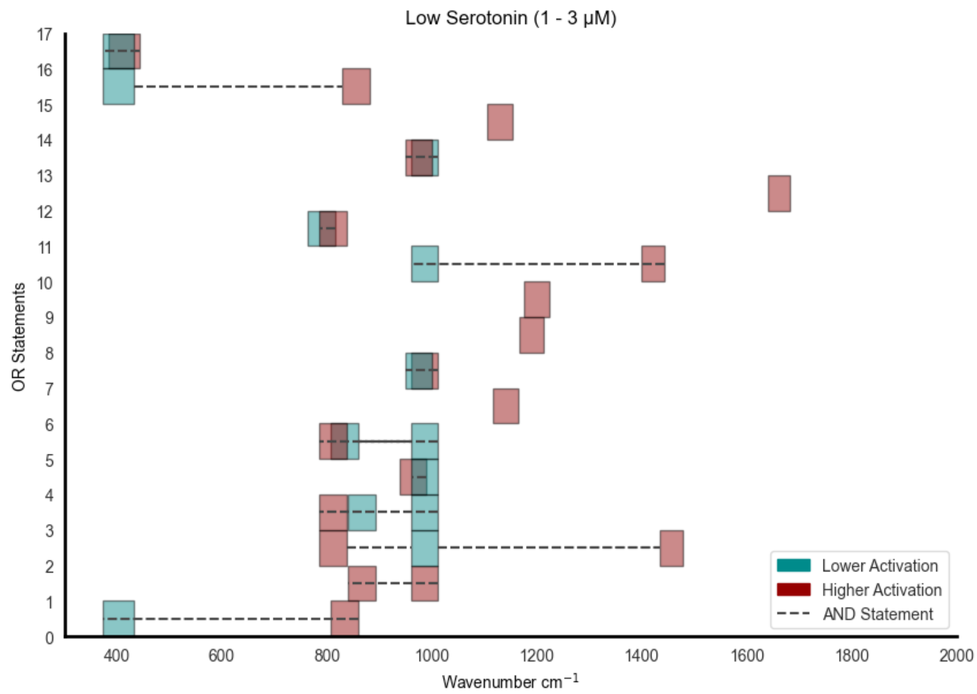

Supplementary Figure 12: **LEN results visualized for low serotonin concentrations.** OR statements are separated vertically, and AND statements are presented level with a dashed line connecting the statements. Blue squares denote lower activation and red squares higher activation.

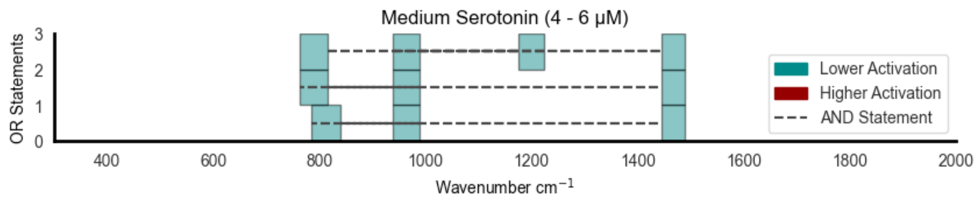

Supplementary Figure 13: **LEN results visualized for medium serotonin concentrations.** OR statements are separated vertically, and AND statements are presented level with a dashed line connecting the statements. Blue squares denote lower activation and red squares higher activation.

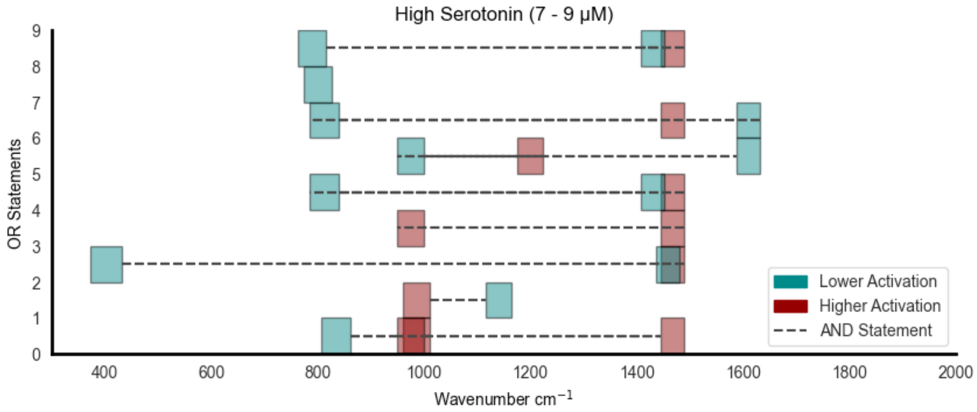

Supplementary Figure 14: **LEN results visualized for high serotonin concentrations.** OR statements are separated vertically, and AND statements are presented level with a dashed line connecting the statements. Blue squares denote lower activation and red squares higher activation.

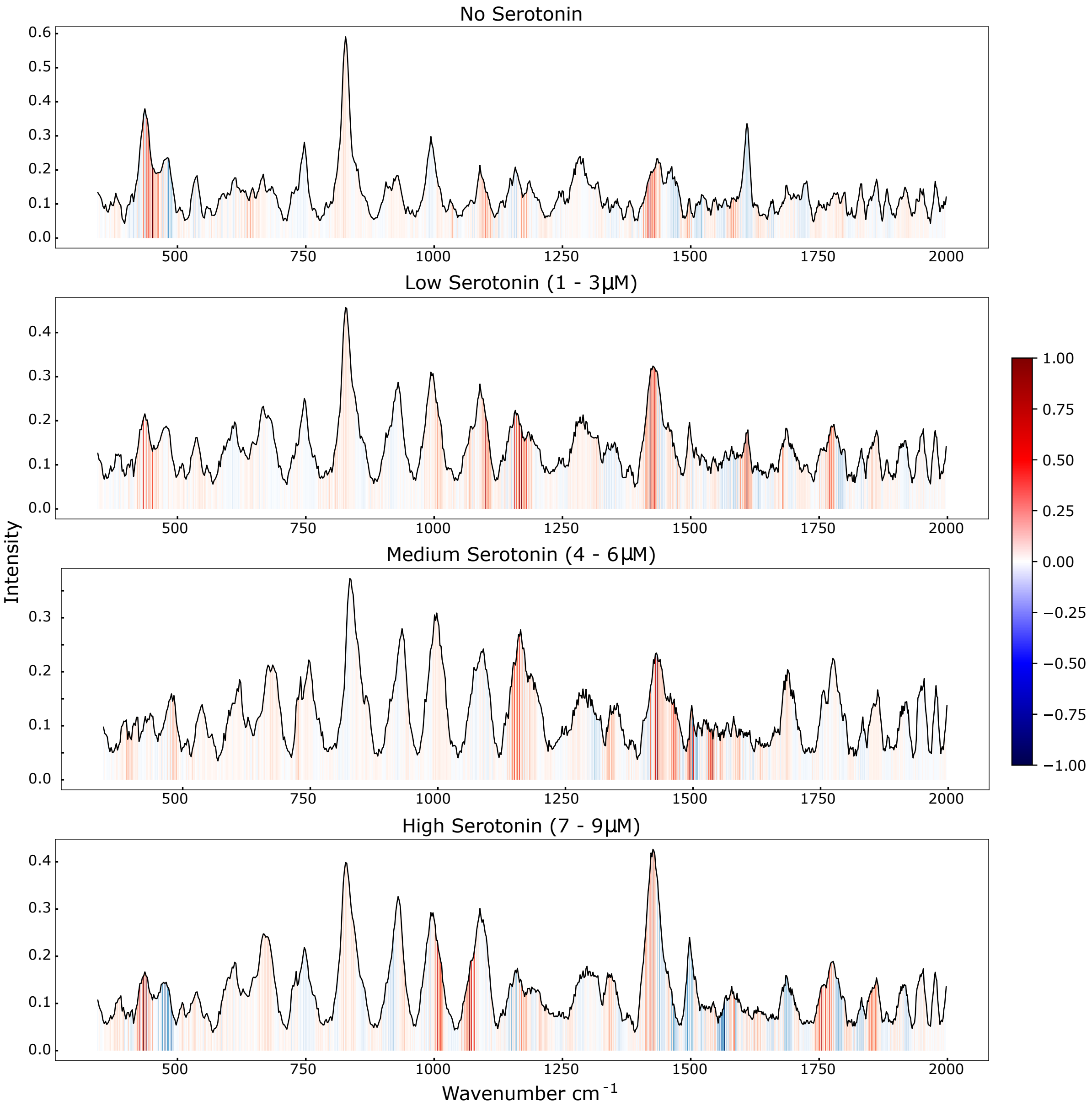

Supplementary Figure 15: **Shapley additive explanations (SHAP) visualized for all serotonin concentration ranges.** Spectra shown are mean spectra across the respective concentration ranges. SHAP values were obtained using Gradient Explainer, and red areas correspond to positive SHAP values and blue areas to negative SHAP values.

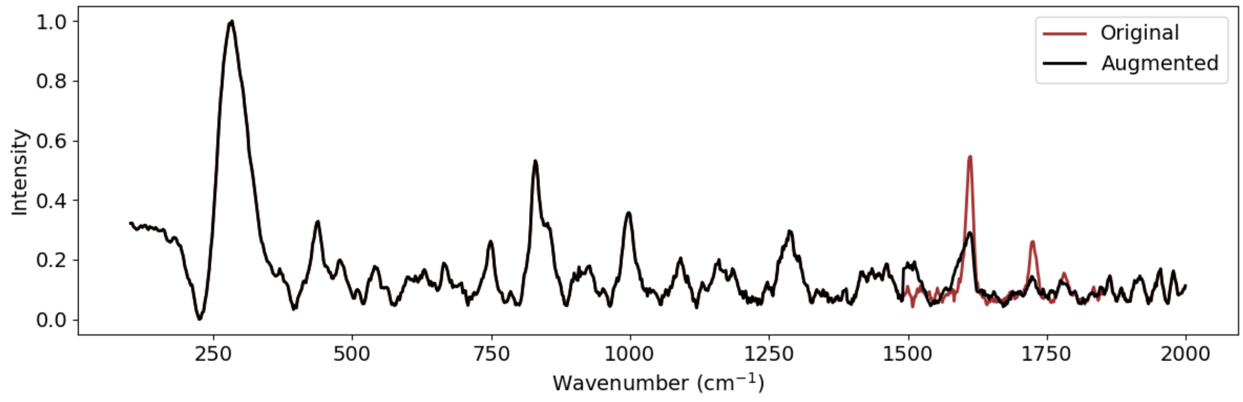

Supplementary Figure 16: **Visualization of the augmentations in the 1 µM serotonin concentration samples.** The original spectra is shown in red, and the augmented spectra in black.

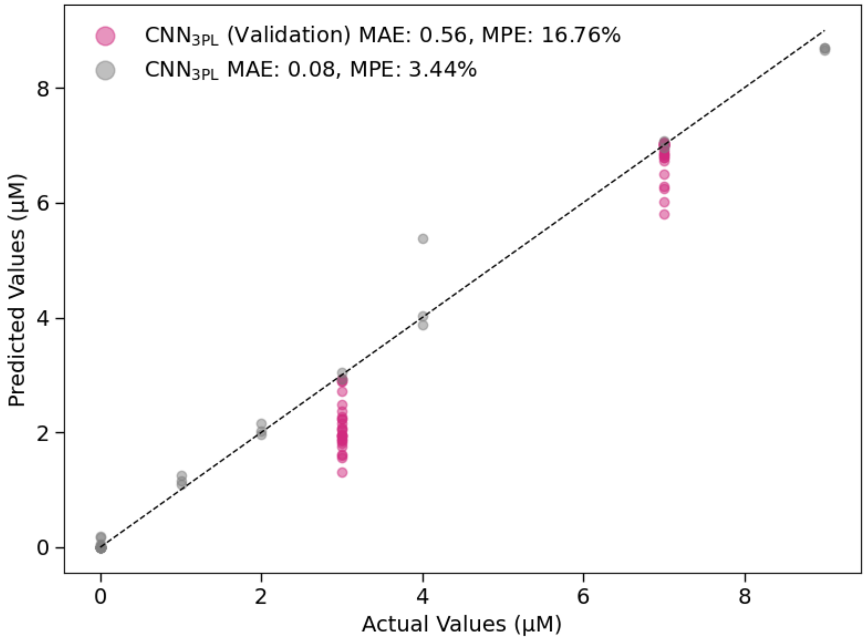

Supplementary Figure 17: **Predictions of the model ensembles on the validation set for the three-parameter logistic output layer CNN model on denoised data, following data augmentation.** Augmentations were made on spectra at 0 and 1 µM serotonin concentrations. MAE = mean absolute error, MPE = mean percentage error.
